# Correction: How Etuaptmumk/Two-Eyed Seeing is used in Indigenous health research: A scoping review

**DOI:** 10.1371/journal.pone.0349369

**Published:** 2026-05-13

**Authors:** The PLOS One Staff

## Notice of Republication

This article was republished on March 24, 2026, to correct to correct an error in the title: the word Indigenous was erroneously left uncapitalized. The publisher apologizes for the errors. Additionally, there were errors in some of the references in the S1 Table. Please download this article again to view the correct version. The originally published, uncorrected article and the republished, corrected articles are provided here for reference.

## Supporting information

S1 File Originally published, uncorrected article. (PDF)

S2 File Republished, corrected article. (PDF)
