# Supplementary material for: How Etuaptmumk/Two-Eyed Seeing is used in Indigenous health research: A scoping review
Source: PLoS One. 2024 Sep 19;19(9):e0310247. doi: 10.1371/journal.pone.0310247 (PMC11412502; doi:10.1371/journal.pone.0310247)
Supplement: S1 Table — Year of publication, study participants, geographical location, research design and aim of study. (DOCX) [file pone.0310247.s002.docx]

# **S1 Table. Characteristics of articles that report using Etuaptmumk/Two-Eyed Seeing*.**

*This table is adapted from a previously published charted table [1] using the criteria of this scoping review.

| **Author and year** | **Research participants and study location** | **Research design** | | **Study objectives** |
| --- | --- | --- | --- | --- |
|  |  | **Empirical: TES; CBPR, CBR, CBPA, PR, or PAR; QT or QL** | **Non-Empirical** |  |
| Ahmed et al. 2023^26^‡  (Goose) | Participants: Fort Albany First Nation male members (e.g., Elders, land experts, youth) aged 18 years or over with a valid Possession and Acquisition License  Study location: James Bay Region, Northern Ontario, CAN | TES, CBPR  QT: Saliva to measure salivary cortisol ElectroChemiLuminescence Immunoassay analysis, paired t-test and sensitivity analysis  QL: Walk along Albany River taught goose hunting and handling, photovoice, semi-directed interviews | N/A | Address the issues of food security and environmental conservation and contribute to the health and well-being of participants and the continent-wide initiative to harvest over-abundant goose species. Also, reconnect Elders and youth in the Niska program to revitalize goose harvesting activities and associated Indigenous knowledge within the community. |
| Ahmed et al. 2023^25^‡  (Beaver) | Participants: Fort Albany First Nation members (e.g., Elders, land experts, youth) aged 18 years or over  Study location: James Bay Region, Northern Ontario, CAN | TES, CBPR  QT: Saliva to measure salivary cortisol ElectroChemi Luminescence Immunoassay analysis, paired t-test  QL: Taught beaver trapping around the community, photovoice, semi-directed interviews | N/A | Reconnect the Elders and youth in the Amisk (beaver) program to revitalize traditional on-the-land activities and beaver harvesting and associated activities within the community; reconnect the youth with the land; protect the community from local flooding; enhance local water quality; and contribute to the well-being of the participants and their community. |
| Auger 2016^69^‡ | Participants: N/A  Location of relevancy: CAN; US | Meta-synthesis of QL research | N/A | Describe and interpret qualitative research on cultural continuity for North American Indigenous Peoples. |
| Barker et al. 2021^28^‡ | Participants: Inuit using tobacco aged 18 years or over, Helpers working with Inuit  Study location: Ottawa (Inuit), across Canada (Helpers), CAN | CBPR, TES  QT: Online questionnaire, editorial edits and suggestions on toolkit  QL: Focus group, engagement circle | N/A | Co-create a culturally relevant and safe toolkit for tobacco addiction treatment reflective of Inuit traditional ways of learning, knowing, healing, and recovery to improve effectiveness of tobacco cessation services for Inuit living in Ontario. |
| Baydala et al. 2016^17^‡ | Participants: Four Maskwacis (Cree) Nations elementary and junior high school students, Elders, parents, school personnel, program facilitators  Study location: Alberta, CAN | CBPR  QT: Sequential longitudinal cohort design, questionnaires; QL: Focus groups | N/A | Culturally adapt the Life Skills Training program to reflect the culture, language, and visual images of the Maskwacis community, deliver it in Maskwacis schools, and evaluate its impact. |
| Benoit et al. 2019^18^‡ | Participants: Cis- or trans-women self-identifying as First Nation, Métis or Inuk, aged 18 years or over  Study locations: Toronto & Thunder Bay, Ontario, CAN | CBR, TES  QT: Questionnaires; QL: Sharing circles or interviews | N/A | Present the discourse on racism experiences of Indigenous women living in two urban Canadian cities. |
| Black et al. 2016^59^‡ | Participants: N/A  Location of relevancy: CAN | N/A | Policy analysis | Increase Indigenous participation in the decision-making process on environmental issues and propose a framework to meet this outcome. |
| Blangy et al. 2018^80^‡ | Participants: N/A  Location of relevancy: Nunavik, Québec, CAN | N/A | Description of OHMi-Nunavik research program | Introduce the OHMi Nunavik Special Issue, present the OHMi Nunavik methodology and review ongoing projects and their contributions to Nunavik community wellbeing. |
| Bruner et al. 2019^39^‡ | Participants: First Nations, Métis or Inuit youth, aged 15-25 years  Study locations: 7 urban and 3 reserves and rural communities, British Columbia, Nunavut, Newfoundland, Labrador, CAN | QL: Sharing circles | N/A | Understand Indigenous youth development within the context of sport and physical activity through the voices, stories, and experiences of Indigenous youth. |
| Building More Bridges Team, 2023^71^‡ | Participants: People aged 18 years or over, initiating combination antiretroviral therapy in or after 2000 and before January 2016  Study location: Regina and Saskatoon, Saskatchewan, CAN | TES  QT: Multiple linear regression analysis – retrospective, geographic information systems methods and forward sortation area; QL: Gathering | N/A | Nurture relationships among a team of researchers  Of mostly Indigenous people living with HIV and to understand the impact of distance to HIV care on HIV outcomes (and markers of quality HIV care) in Saskatchewan using CANOC. |
| Butler et al. 2018^95^‡ | Participants: N/A  Location of relevancy: University of Saskatchewan, Saskatchewan, CAN | N/A | Discussion of Indigenous engagement | Describe implementing the "learn where you live" delivery model at the universities College of Nursing by the Strategist for Outreach and Indigenous Engagement. |
| Cabrera et al. 2015^19^‡ | Participants: Talthan community  Study location: British Columbia, CAN | CBPR  QL: Focus groups, interviews | N/A | Explore the impact and interplay of medicalization with the Nation’s knowledge and approaches to wellness in relation to early onset familial Alzheimer disease. |
| Carter et al. 2017^74^‡ | Participants: First Nations men in Toronto  Study location: Ontario, CAN | TES  QL: Narrative methodology, interviews | N/A | Counter negative Aboriginal men stereotypes by exploring positive Aboriginal identity narratives and explore ways health care providers deliver culturally safe care and support Aboriginal clients’ wellbeing. |
| Castleden et al. 2017^57^‡ | Participants: Community-based and academic researchers conducting water research using Western and Indigenous knowledge systems  Study locations: CAN | QL: Semi-structured interviews | N/A | Examine how different ontologies of water are part of different ways of relating to water and how water has an important effect on the allocation and enactment of roles in collaborative research partnerships. |
| Chatwood et al. 2015^75^‡ | Participants: N/A  Location of relevancy: Fly-in lodge in northern CAN | N/A | Discussion of research method | Describe a collaborative mixed method study with Indigenous knowledge used by researchers and Indigenous knowledge holders on Indigenous values underlying health systems stewardship. |
| Clark 2014^98^‡ | Participants: Inuit recommended or endorsed by the Manitoba Urban Inuit Association, electronic health program managers, Inuk Elder  Study location: Manitoba, CAN | TES  QL: Focus groups, interviews | N/A | Offer best practice recommendations for how to communicate personal health information services to Manitoba Inuit and increase their awareness of issues related to electronic health information within the healthcare system. |
| Crooks et al. 2018^23^‡ | Participants: MHFAFN facilitators and participants from First Nations communities, organizations in urban centres, & rural communities  Study location: 4 provinces, CAN | CBPR, TES  QT: Surveys; QL: Conversational interviews | N/A | Undertake a feasibility study of the Mental Health First Aid First Nations (MHFAFN) course to assess its acceptability, cultural adaptation, and preliminary participant outcomes. |
| Danto et al. 2021^89^‡ | Participants: Community members known to each other  Study location: Mushkegowuk Territory, West Coast James Bay, Ontario, CAN | TES  QL: Indigenous methodology, interviews and member checking | N/A | Understand shared and distinct features of healing across communities for enriching knowledge transmission to future generations and identifying important practices and traditions for those involved in providing mental health services for Indigenous people. |
| Denny et al. 2016^67^‡ | Participants: N/A  Location of relevancy: Nova Scotia, CAN | N/A | Discussion of salmon governance | Compare conservation concepts in Atlantic salmon management from a non-Aboriginal state perspective with a Mi’kmaq perspective to develop Atlantic salmon governance initiatives. |
| Fayed et al. 2018^63^‡ | Participants: N/A  Author location: British Columbia, CAN | N/A | TES  Reconciliation-based analysis, literature review | Expose the colonial etiology of hepatitis C infection in Canada and propose potential anti-colonial approaches to hepatitis C wellness and health care for Indigenous people. |
| Fontaine et al. 2019^93^‡ | Participants: First Nations women aged greater or equal to 65 years with a medically diagnosed condition or been a caregiver for someone with a heart condition  Study locations: Winnipeg and Northern communities, Manitoba, CAN | QL: Digital storytelling, learning circles | N/A | Identify concepts, language, and experiences of heart health among First Nations women. |
| Galway et al. 2022^29^‡ | Participants: Community Knowledge Holders (Elders, Knowledge Keepers, community members) with ties to the land of diverse age, gender and land relationships  Study location: Fort William First Nation, Northern Ontario, CAN | CBR, TES  QL: Semi-structured conversational interview | N/A | Explore the connections among climate change, land, and health, drawing on the perspectives of Community Knowledge Holders and members with ties to the land in Fort William First Nation. |
| Gray et al. 2019^104^‡ | Participants: First Nations communities  Study location: New Brunswick, CAN | QT: Contaminant screening via analytical chemistry | N/A | Assess spatial and temporal environmental contamination in muskrat root; assess human health risk by consumption; and provide a baseline against which future monitoring could be compared with. |
| Gresku et al. 2022^81^‡ | Participants: Indigenous communities  Location of relevancy: North America | N/A | TES  Narrative review | Inform and support the co-creation of an evaluation framework for two interconnected, ongoing, community-based, Indigenous-led health studies. |
| Hall et al 2015^73^‡ | Participants: N/A  Study locations: 12 First Nations treatment centres across CAN | N/A | Discussion of TES in a research study | Apply TES in the 1^st^ year of a 3-year study on the effectiveness of cultural interventions in First Nations alcohol and drug treatment in Canada. |
| Hall 2015^49^† | Participants: N/A  Study locations: 12 community treatment centres in CAN | N/A | Interview transcription | Describe trans-disciplinarity, its relationship to Indigenous ways of knowing; and using TES in addictions research with Indigenous peoples. |
| Hatala et al. 2017^45^‡ | Participants: Plains Cree and Métis youth, aged 15-25 years  Study location: Saskatoon, Saskatchewan, CAN | TES  QL: Interviews | N/A | Determine how youth experience or orient themselves toward conceptions of time; the impact of systemic social inequities and historical or contemporary traumas; and individual strategies and interpersonal contexts that support processes of resilience and wellbeing. |
| Hatala et al. 2020^60^‡ | Participants: Plains Cree and Métis youth, aged 15-25 years  Study location: Inner city neighbourhoods, Saskatoon, Saskatchewan, CAN | TES  QL: Photovoice, interviews & talking circles | N/A | Explore how connections to land and nature could inform public health interventions for contemporary Indigenous young people experiencing various social inequities within their urban environments. |
| Hovey et al. 2017^68^‡ | Participants: N/A  Location of relevancy: Kahnawake Mohawk Territory, Québec, CAN | N/A | Discussion of TES in a research study | Bring together a health promotion research team to understand shared Indigenous & non-Indigenous perspectives of diabetes prevention and health promotion in an Indigenous community; show how allied health promotion and Indigenous researchers work together through TES; and describe becoming TES researchers. |
| Hunt et al. 2018^54^‡ | Participants: First Nations youth playing recreational hockey, their parents, and coaches  Study location: First Nations reserve, Northern Ontario, CAN | TES  QT: Surveys | N/A | Begin to understand concussion in youth hockey in a First Nations community in Canada and to determine the impact of a novel concussion education workshop. |
| Hutt-MacLeod et al. 2019^79^‡ | Participants: N/A  Location of relevancy: Eskasoni, Nova Scotia, CAN | N/A | Review of a mental health service | Describe implementing the ACCESS OM objectives for youth mental health service transformation within a pre-existing Fish Net Model of transformative youth mental healthcare service in Eskasoni. |
| Jacklin et al. 2020^30^‡ | Participants: Expert Anishinaabe Language Group aged 55-68 years (men and women) who understand traditional and contemporary changes to language, communicate in English and Anishinaabemwin and respected community standing; participants in validity pilots (Pilot 1: aged 45-60 years and aged 61-80/ Pilot 2: aged 45-70); health professional group servicing 7 First Nation communities nurses, an occupational therapist, a physician, and home care and personal support workers working with older adults or conducting cognitive assessments  Study location: Seven Anishinaabe First Nations communities, Manitoulin Island region, northeastern Ontario, Canada | CBPR, TES  QL: Meetings and consultations (EALG, Knowledge Keeper [Elder] and KICA authors), interview/consultation (health professionals) | N/A | Adapt the Kimberly Indigenous Cognitive Assessment (KICA) for use as a brief cognitive test with Anishinaabe populations in Canada |
| Kurtz et al. 2017^44^‡ | Participants: Indigenous nursing new graduates, employers, educators, and policy makers  Study location: Western CAN | TES  QL: Indigenous methodology: interviews, one day forum, talking circles | N/A | Examine Indigenous nurses’ transition from education to practice and their career development experiences; increase understanding retention issues for Indigenous nurses in the workplace. |
| Latimer et al. 2014^61^‡ | Participants: N/A  Author location: CAN | N/A | Literature review | Raise awareness and create new understandings in pediatric pain knowledge to reflect the best of Indigenous and Western perspectives on how to enhance health care encounters, reduce the hurt & increase Aboriginal children wellbeing. |
| Latimer et al. 2018^20^‡ | Participants: First Nation youth aged 8-17 years  Study locations: 4 First Nations communities (3 Mi'kmaw and 1 Wolastoqey regions), New Brunswick, Nova Scotia, Prince Edward Island; CAN | CBPA, TES  QL: Ethnographic techniques, talking circles, art workshop | N/A | Determine how First Nations youth express pain to inform culturally appropriate assessment and treatment. |
| Latimer et al. 2020^38^‡ | Participants: First Nations children and youth aged 8-17 years, parent, Elders, and professionals practicing in the community  Study location: Mi’kmaw and Wolastoqey nations, Eastern CAN | CBPA, TES  QT: Demographic questionnaire; QL: Semi-structured conversational sessions and interviews | N/A | Gather the healthcare experiences, pain perspectives, and the strategies to improve the healthcare encounter for First Nations children and youth and health providers from four First Nations communities. |
| Mantyka-Pringle et al. 2017^48^‡ | Participants: First Nations and Métis communities  Study location: Slave River Delta, Northwest Territories, CAN | TES  QL: Field observations, document reviews, interviews | N/A | Present a TES approach for co-producing and blending knowledge about ecosystem health by using an adapted Bayesian Belief Network for the Slave River and Delta region in Canada's Northwest Territories. |
| Marsh, Coholic, et al. 2015^77^‡ | Participants: N/A  Location of relevancy: Ontario, CAN | N/A | Literature review | Explore the feasibility of blending Aboriginal healing practices with a Western treatment model to address intergenerational trauma with substance use disorders in Aboriginal peoples using TES. |
| Marsh, Cote-Meek, et al. 2015b^40^‡ | Participants: N/A  Author location: Sudbury, Ontario, CAN | N/A | Review of research process | Describe the research process taken to incorporate TES Indigenous decolonizing methodology into the treatment of intergenerational trauma and substance use disorders in Aboriginal peoples, using the Seeking Safety model. |
| Marsh et al. 2016^58^‡ | Participants: Indigenous males and females of Ojibway, Cree, and Métis heritage; aged 24-68 years; had intergenerational trauma and substance use disorder  Study location: Sudbury, Ontario, CAN | TES  QT: Questionnaires; QL: Sharing circles, semi-structured interviews, discussion meetings | N/A | Explore whether the blending of Indigenous traditional healing practices and the Western treatment model Seeking Safety, used to treat post-traumatic stress disorder and substance use disorders, resulted in a reduction of intergenerational trauma symptoms and substance use disorders. |
| Marsh et al. 2018^78^‡ | Participants: Males and females of Ojibway, Cree, and Métis heritage; aged 24-68 years; had intergenerational trauma and substance use disorder  Study location: Sudbury, Ontario, CAN | TES  QL: Indigenous decolonizing methodology, sharing circles, semi-structured interviews | N/A | Explore the application of the sweat lodge ceremony as a component of Indigenous Healing and Seeking Safety. |
| Marsh et al. 2020^47^‡ | Participants: Male and female who are Indigenous facilitators that worked with Indigenous people experiencing intergenerational trauma and substance use disorder and have an understanding of or experiences of one or both  Study location: Sudbury, Ontario, CAN (women) | TES,  QL: Sharing circles, semi-structured interview | N/A | Explore the growth and changes the training of Indigenous facilitators for a TES research treatment intervention for intergenerational trauma and addiction brought about for, as well as their perception of how their changes impacted their clients. |
| Martin et al. 2017^43^‡ | Participants: Elders, key informants (health care providers, family caregivers, community leaders)  Study locations: Little Saskatchewan First Nation, Manitoba, CAN | TES  QL: Participatory framework, critical ethnography interviews | N/A | describe the merits and challenges of using TES (Elders and typically non-Indigenous decision-makers) to guide a collaborative research project with a First Nation community in Manitoba, Canada devastated by a human-made flood. |
| McKivett et al. 2020^62^‡ | Participants: N/A  Location of relevancy: AUS | N/A | Discussion on approach to medical education research | Explore the TES approach in the context of Indigenous medical education research and draws on an Australian research project as an example of its application. |
| McMillan et al. 2016^56^‡ | Participants: N/A  Location of relevancy: Atlantic CAN | N/A | Discussion of fishery management | Review recent developments on fishery policy and management processes where the Mi'kmaw are working to revitalize the place of netukukimk. |
| Njeze et al. 2020^53^‡ | Participants: Indigenous male and female youth from nêhiyaw (Plains Cree, Dene, Métis aged 16-24 years  Study location: Saskatchewan, CAN | TES  QL: Seasonal sharing circles, individual conversational interviews, photovoice, naturalistic interactions (participant observations) | N/A | Identifies processes of resilience among urban Indigenous youth as it relates to how individual, social, and cultural processes overlap and inter- sect in important ways to support resilience and overall wellness. |
| Peltier 2018^42^‡ | Participants: N/A  Location of relevancy: First Nations communities, Manitoulin Island, Ontario, CAN | N/A | Discussion of TES in a research study | Add to existing qualitative methods by applying TES in the bridging of Indigenous and participatory methodologies. |
| Pilarinos et al. 2023^85^‡ | Participants: Indigenous people accessing urban health care services of Vancouver Coastal Health aged 18 years or over  Study location: Vancouver, Richmond, North Vancouver CAN | TES  QL: Sharing circles | N/A | Understand Indigenous patients' experiences with racism and improving cultural safety within health care in Vancouver, Canada. |
| Poirier and Tait-Neufeld 2023^31^‡ | Participants: Community leaders involved with day-to-day food-related activities in community (i.e., women, Elders)  Study location: Coast Salish Community, Vancouver Island, CAN | CBPR, TES  QL: Indigenous methodologies, informal conversations, morning talks while hiking, sharing circle | N/A | Explore perspectives and experiences (e.g., barriers and facilitators) of Indigenous food sovereignty (IFS) efforts on Vancouver Island. |
| Quinn 2022^46^‡ | Participants: Indigenous adults aged 27-69 years, completed grade 8 education, former youth in care, defined as successful (participating in school, employment, community volunteer work or leadership, and/or parenting)  Study location: Reserves, rural and urban areas, Ontario, CAN | TES  QL: Traditional and constructivist grounded theory integrated in the Relational Worldview Method and decolonizing Indigenous research epistemologies; Interview | N/A | Explore the experiences of intergenerational trauma, cultural connections, and identity among Indigenous former youth in child welfare care of the Ontario government. |
| Rand 2016^24^‡ | Participants: Inuit women aged 18-61 years  Study location: Kugluktuk, Nunavut, CAN | CBPR, TES  QL: Storytelling sessions | N/A | Create a dialogue with Inuit women to address the lack of information to inform programming to improve their sexual health, their families, and their communities in the Canadian Arctic. |
| Roburn, 2021^90^‡ | Participants: Kwawaka'wakw First Nations salmon fishers  Study location: Kwawaka'wakw territory, Broughton Archipelago, British Columbia, CAN | QL: Observations of select social media forums (formal and informal) (i.e., Facebook, Instagram) | N/A | Qualitative examination of social media and media communications of Kwawaka'wakw around salmon farming. |
| Rowan et al. 2015^50^‡ | Participants: N/A  Author location: CAN | N/A | Scoping review | Identify and map literature on cultural interventions in addictions treatment; and describe the nature, extent, and gaps in the literature. |
| Rowett 2018^96^‡ | Participants: N/A  Location of relevancy: Mi’kmaw and Wolastoqey nations | N/A | Discussion of TES as a research approach | Describe Etuaptmumk and its contribution to relationality and relational accountability in future research; review its usage in the literature; and describe the author’s internalized ways of knowing, specifically Mi'kmaw & Wolastoqey knowledges. |
| Sam et al. 2022^82^‡ | Participants: Indigenous adolescent women, men and Two Spirit, aged 20 to 24 years  Study location: Urban centre, Southern British Columbia, CAN | QT: eHeals tool  QL: Design circles | N/A | Understand how we might Indigenize an eMental health screening app for adolescents accessing primary health  care delivered in an integrated clinic setting. |
| Saskamoose et al. 2017^76^‡ | Participants: First Nations community members, cultural experts (e.g., Elders, medicine people, ceremonialists, or herbalists)  Study location: Saskatchewan, CAN | QL: Indigenous-centred research process, in-depth Indigenous qualitative consultations | N/A | Describe the theoretical development of the Indigenous Cultural Responsiveness Theory, a decolonizing model that aims to improve First Nations well-being in Saskatchewan. |
| Shrivastava et al. 2020a^86^‡ | Participants: Care providers, administrators, Cree patients  Study location: Four Eastern James Bay Cree communities in Northern Québec, CAN | PR, TES  QL: Group discussions, individual interviews | N/A | Contribute to the deep understanding of the cultural aspects of the integration of oral health into primary health care at an Indigenous health organization. |
| Shrivastava et al. 2020b^92^‡ | Participants: N/A  Study location: AUS, North America | N/A | Scoping review | Systematically map the available programs and their outcomes on the integrated primary oral health care  programs in Indigenous communities underpinned by  the two-eyed seeing concept. |
| Sivertsen et al. 2020^65^‡ | Participants: Aboriginal residents in residential aged care and carers  Study location: AUS | QL: Aboriginal centred research, interpretive descriptive approach, semi-structured interviews using a conversational ‘yarning’ style | N/A | Investigate how the Aboriginal residents’ spiritual well-being related to living in residential aged care in South Australia. |
| Snooks et al. 2021^32^‡ | Participants: N/A  Study location: Northern Ontario, CAN | PAR | Critical reflection | Reflection on multiyear collaborative research project based on a commitment to feminist, decolonial, and Indigenous methodologies and the use of PAR with community-based service providers and persons with lived experience in human trafficking and the sex industry more generally. |
| Stelkia et al. 2020^83^‡ | Participants: First Nations Knowledge Keepers, youth from Stó:lō communities  Study location: Fraser Salish region, British Columbia, CAN | TES  QL: Sharing circle | N/A | Explore what connection to land, water, and territory means for health and wellness for First Nations in the Fraser Salish region in British Columbia, Canada. |
| Sylliboy 2021^33^‡ | Participants: Two Spirit people  Study location: Wabanaki Two-Spirit Alliance, Atlantic CAN | CBR, TES  QT: Online demographic and self-reported health forms; QL: Guided interviews | N/A | Explore Two-spirit stories and narratives about the coming out process for Two-spirit people from the cultural perspectives of Two-spirit Mi’kmaw indivudals (one of them being the author). |
| Tarasuk et al. 2021^52^‡ | Participants: First Nations and Métis people and Inuit who inject drugs  Study location: 14 Sentinel sites across CAN | TES  QT: Interview-administered questionnaire, biological samples (HIV and hepatitis C blood spot testing), descriptive statistics | N/A | Present national surveillance findings among Indigenous participants from Phase 4 of the Tracks survey of PWID in Canada, conducted between January 1, 2017 and May 9, 2019, at participating sentinel sites in Canada. |
| VanEvery et al. 2022^34^‡ | Participants: Elders or Knowledge Holders and health clinicians (experienced working with Indigenous youth in the area), youth (self-identified Indigenous, aged 13 years or over)  Study location: Aboriginal Health Centre serving two urban communities in Brantford and Hamilton, Southern Ontario, Iroquois Territory, CAN | CBR, PAR, TES  QL: One-on-one interviews, small group conversation sessions | N/A | Mobilize Indigenous knowledge to improve the health care experiences for urban Indigenous youth. More specifically, to understand how Indigenous youth describe, experience, manage pain and hurt and how they seek care. |
| VanEvery and Wright 2021^91^‡ | Participants: Indigenous mothers, primary care providers, providers of early child development services  Study location: Hamilton, Ontario, CAN | TES  QL: Interviews | N/A | Describe how mothers experience the complexities of supporting the development of their infants’ Indigenous identities within an urban, off-reserve environment as it relates to their health and well- being. |
| Vorobyova et al. 2022^35^‡ | Participants: Older adults living with HIV aged 50 years or over with present, past, required, or denied use of home and community care services within Vancouver Coastal Health Authority  Study location: Vancouver, British Columbia, CAN | CBR, TES  QL: Semi-structured interviews | N/A | Identify barriers and facilitators experienced by older adults living with HIV accessing home and community care. |
| Venner et al. 2018^94^‡ | Participants: American Indians, Alaskan Natives  Location of relevancy: US | QL: One-day meeting | N/A | Describe the results of a meeting of key stakeholders to elicit feedback on the acceptability and uptake of medication assisted treatment for opioid use disorders among American Indians and Alaskan Natives. |
| Victor et al. 2019^70^‡ | Participants: Blackfoot who are homeless and use substances, and key informants (program facilitators and individuals interacting regularly with participants)  Study location: Alberta, CAN | TES  QL: General inductive approach to impact and process evaluation, semi-structured interviews | N/A | Evaluate a program for Indigenous people experiencing homelessness and substance misuse. |
| Vukic et al. 2012^72^‡ | Participants: N/A  Location of relevancy: CAN | N/A | Discussion of nursing research method | Explain how CBPR and the principles of OCAP help to integrate TES and ethical space in shaping nursing research to address health priorities with Aboriginal peoples. |
| Vukic et al. 2016^22^‡ | Participants: Mi’kmaw youth and Elders  Study location: rural community (Mimikej as pseudoname), Nova Scotia, CAN | CBPR, TES  QL: Talking circles, community forum, storytelling, field notes, participant observation | N/A | Understand Mi’kmaw youth mental health. |
| Ward et al. 2023a^36^‡ | Participants: Family caregivers, health and community providers, and healthcare and community leaders  Study location: Samson Cree Nation, (Nîpisîhkopâhk), Enoch Cree Nation (Maskêkosihk), Alberta, CAN | TES, PAR  QL: Semi-structured interviews | N/A | Report the recommendations of family caregivers, providers, and leaders for supporting First Nations family caregivers and the health and community providers employed in First Nations communities. |
| Ward et al. 2023b^37^‡ | Participants: Family caregivers, health and community providers, and healthcare and community leaders  Study location: Samson Cree Nation, (Nîpisîhkopâhk), Enoch Cree Nation (Maskêkosihk), Alberta, CAN | PAR, TES  QL: Interviews | N/A | Understand how family caregivers, health and community providers, and leaders experience care and support for the family caregivers in two Alberta First Nations Communities. |
| Webkamigad et al. 2020^27^‡ | Participants: Health Canada’s First Nations and Inuit Home and Community Care program managers  Study location: CAN | CBPR, TES  QL: Review of previously collected interview data, on-going meetings with stakeholder | Literature review, environmental scan of relevant unpublished web-based documents and paper-based resources | Outline a unique and innovative process resulting in two evidence-based Indigenous-specific fact sheets on dementia care for caregivers, older adults, people with dementia, and community health care providers. |
| Wekerle et al. 2022^84^‡ | Participants: Indigenous Health care and social service providers (HSSP; women held some roles as a Cultural Leader, Knowledge Keeper or Cultural Holder), serving on- and off- reserve members, aged 18 years and over  Study location: Health care and social service organizations on Six Nations of the Grand River Communities (Seneca (Onondowahgah), Cayuga (Guyohkohnyoh), Onondaga (Onundagaono), Oneida (Onayotekaono), Mohawk (Kanienjahagen), and Tuscarora (Ska-Ruh-Reh) nations), Ontario, CAN | TES  QL: Semi-structured one-on-one interviews | N/A | Describe Six Nations HSSPs perceptions of the value and relevance of the Creating Safety module for HSSPs working with Indigenous clients; and identify and describe adaptations needed to improve the module’s ability to support HSSPs to be able to provide culturally, physically, and emotionally safe clinical encounters with Indigenous clients. |
| Whiting et al. 2018^64^‡ | Participants: Eagle Moon Health Office Team including Indigenous and non-Indigenous health consultants/ practitioners (Knowledge Keepers, healers, Elders, administrators)  Study location: Qu’Appelle Health Region, Regina, Saskatchewan, CAN | TES  QL: Exploratory research, interpretive descriptive approach | N/A | Explore the perspectives/experiences of select Saskatchewan health professionals practicing across diverse Indigenous and mainstream healthcare services to understand the concept from their perspectives. |
| Whitty-Rogers et al. 2016^21^‡ | Participants: Mi’kmaq women with gestational diabetes mellitus  Study location: 2 Mi’kmaq communities, Nova Scotia, CAN | PAR, TES  QL: One-on-one conversational interviews, talking circles | N/A | Explore and gain insight into the experiences of Mi’kmaq women with gestational diabetes mellitus and explore how social determinants of health and existing health policies shape these experiences. |
| Wolfson et al. 2019^97^‡ | Participants: Community leaders, frontline workers, policymakers, and researchers  Study location: Unceded Territories of the Coast Salish Peoples and the Musqueam Territory, CAN | TES  QL: One-day meeting and a workshop | N/A | Describe the consensus statement and exemplary fetal alcohol syndrome disorders (FASD) prevention programs from Indigenous communities and organizations across Canada to highlight identity, culture, and relationships as central elements of FASD prevention in Indigenous communities. |
| Wright et al. 2019a^55^‡ | Participants: N/A  Study location: Hamilton, Ontario, CAN | N/A | Discussion of TES in research | Describe the application of TES to a community-engaged study aimed at understanding how Indigenous mothers experience using health care to meet the health needs of their infants. |
| Wright et al. 2019b^66^‡ | Participants: Indigenous mothers of infants aged less than 2 years, providers of early childhood development services  Study location: Ontario, CAN | TES  QL: Interpretive description methodology, semi-structured interviews | N/A | This article is part of a larger study exploring the phenomenon of how Indigenous mothers living in the city of Hamilton, Ontario, experience selecting and using health services to meet the health needs of their infants aged less than two years. |
| Wright et al. 2019c^87^‡ | Participants: Indigenous mothers self‐identifying as First Nations, Métis and/or Inuit, living in Hamilton and caring for an infant aged less than 2 years  Study location: Ontario, CAN | TES  QL: Interpretive description methodology, interviews, discussion group | N/A | Develop an understanding of how Indigenous mothers experience selecting and using health services for their infants can assist nurses in improving their access to care. |
| Wright et al. 2019d^88^‡ | Participants: Indigenous mothers and primary care providers  Study location: Ontario, CAN | TES  QL: Interpretive description methodology, semi-structured interviews, discussion group | N/A | Understand how Indigenous mothers—typically responsible for the health of their infants—living in urban areas, experience selecting and using health services to meet the health needs of their infants. |
| Wright et al. 2020^41^‡ | Participants: Indigenous mothers of infants aged less than 2 years, accessing and using the health care system for their infants  Study location: Ontario, CAN | TES  QL: Interpretive description methodology, interviews, discussion group | N/A | Understand how Indigenous mothers experience accessing and using the health care system for their infants. |
| Wright et al. 2021^51^‡ | Participants: First Nations and Métis, and Inuit mothers caring for an infant aged less than 2 years  Study location: Hamilton, Ontario, CAN | QL: Semi-structured interviews, group discussions | N/A | Understand the experience of Indigenous mothers who use web-based information to support the health of their infants. |

Grey literature and journal articles were included: book chapter†, journal article‡.

Abbreviations: Australia = AUS; Canada = CAN; Community-based participatory research = CBPR; Community-based participatory action methodology = CBPA; Community-based research = CBR; Not applicable = N/A; Ownership, control, access, and possession = OCAP; Participatory action research = PAR; Participatory research = PR; Qualitative = QL; Quantitative = QT; Two-Eyed Seeing = TES; United States = US
