# Supplementary material for: How Etuaptmumk/Two-Eyed Seeing is used in Indigenous health research: A scoping review
Source: PLoS One. 2024 Sep 19;19(9):e0310247. doi: 10.1371/journal.pone.0310247 (PMC11412502; doi:10.1371/journal.pone.0310247)
Supplement: S1 Protocol — (DOCX) [file pone.0310247.s003.docx]

Two-Eyed Seeing in Indigenous Health: a scoping review protocol

# **Review Question**

1. How has the concept of ‘Two-Eyed Seeing’ evolved over time?
2. In what ways has ‘Two-Eyed Seeing’ been used in Indigenous health research?

# **Keywords**

# Indigenous peoples; Two-Eyed Seeing; Indigenous Health Research

# **Inclusion Criteria**

### **Participants**

### This review will consider studies that include First Nations, Inuit and Métis people in Canada as study participants. When non-Indigenous populations are involved, such as non-Indigenous health practitioners, the literature will be eligible for inclusion if the research aim or phenomenon of interest is closely related to Indigenous health.

### **Concept**

### Two-Eyed Seeing is a guiding principle that is used to describe the process of bringing together the strengths of Indigenous and Western knowledges for the benefit of all (Hatcher et al. 2009). The term “Etuaptmumk” has been used interchangeably with TES in literature.

### **Context**

### This review will focus on Two-Eyed Seeing in the context of Indigenous Health Research in Canada. The review will utilize CIHR’s definition of Indigenous Health Research:

*“Indigenous Health Research (IHR) can be defined by any field or discipline related to health and/or wellness that is conducted by, grounded in, or engaged with, First Nations, Inuit or Métis communities, societies or individuals and their wisdom, cultures, experiences or knowledge systems, as expressed in their dynamic forms, past and present.*

*Indigenous health and wellness research embraces the intellectual, physical, emotional and/or spiritual dimensions of knowledge in creative and interconnected relationships with people, places and the natural environment. Such research is based on the right to respectful engagement and equitable opportunities; it honours culture, language, history, and traditions.*

*Indigenous health and wellness research, thus defined, may be implemented and adapted in research involving Indigenous peoples around the world. Whatever the methodologies or perspectives that apply in a given context, researchers who conduct Indigenous research, whether they are Indigenous or non-Indigenous themselves, commit to respectful relationships with Indigenous peoples and communities” (http://www.cihr-irsc.gc.ca/e/50340.html)*

### **Types of Sources**

### This scoping review will consider all existing literature that fits the inclusion criteria. Eligible sources of evidence will include: primary research of all study designs, reviews and meta-analyses, text and opinion papers, theses and dissertations, conference proceedings, newsletters, and publications by Canadian government/Indigenous organizations.

# **Methods**

This scoping review will follow the six-step approach proposed by Arksey and O’Malley (2005): identify the research question; searching for relevant studies; selecting studies; charting the data; collating, summarizing, and reporting the results; together with an optional consultation exercise. Where necessary we will seek clarification on the methodology by Arksey and O’Malley by consulting with Levac et al. 2010 manuscripts on advancing the methodology.

### **Search Strategy**

A three-step search strategy will be conducted through consultation with an experienced JBI systemic review library scientist. The initial search will be undertaken in CINAHL and PubMed followed by analysis of text words contained in the title and abstract along with index terms used to describe the article. A second search will then be conducted using all identified keywords and search terms across all included databases. Thirdly, the reference lists of all identified articles will be searched for additional record. Key journals related to Indigenous health will be hand searched for eligible studies. This study will only include articles after 2004 since this is the year that Two-Eyed Seeing was first introduced to the academic community by Elders Albert and Murdena Marshall. This study will only include articles written in English.

### **Information Sources**

### The databases to be searched will include: PubMed, Academic Search Premier, PsyINFO, CINAHL, Bibliography of Native North American, EMBASE, PoQuest Dissertations and Theses, and Indigenous Studies Portal. Searches for grey literature will be conducted on the website of the Institute for Integrative Science & Health (IISH). In addition, the following key journals will be hand-searched for eligible studies: International Journal of Indigenous Health, International Journal of Circumpolar Health, and *Pimatisiwin*: A Journal of Aboriginal and Indigenous Community Health.

**Study Selection**

All identified studies will be entered into EndNote and duplicates across databases will be removed automatically. These citations (without duplicates) will then be imported to Covidence – an online systematic review platform. Two reviewers will complete title and abstract screening independently, selecting studies against the pre-defined inclusion criteria. Following this step, the full text of eligible studies will be retrieved and uploaded to Covidence. Two reviewers will independently assess the eligibility of full-text articles and provide a rationale for exclusion, which will be listed in an appendix of the final review. Throughout the study selection phase, discrepancies between two reviewers will be settled through discussion or possibly a third reviewer, if required. In the final review, the study selection process will be summarized in a PRISMA flowchart.

### **Data Extraction**

### The following details will be extracted from included studies:

### Author(s)

### Year of publication

### Type of literature, e.g., journal articles, theses and dissertations, reports and others

### Goal of the study

### Study participants

### Geographical location of study

### Area of health research, e.g., nursing, public health, geography, multidisciplinary, medicine

### Definition of TES

### Usage of TES, i.e., where during the research process TES was used

### Main findings

### Author’s conclusion

### To begin with, two reviewers will trial the charting table on one article and compare results. The remaining data extraction will be completed independently by two reviewers, and discrepancies will be resolved through discussion or a third reviewer, if required. As data extraction continues, the charting table will likely be updated, and the final version will be presented in the report as an appendix.

### **Presentation of Findings**

### Main findings will be summarized in tabular forms accompanied by narratives and figures. Tables will be used to compare different definitions and usages of Two-Eyed Seeing accompanied by narrative analysis. As the review continues, other themes will likely emerge from the literature and be presented in the final report.

# **Conflicts of Interest**

The authors declare no conflict of interest.
