# Supplementary material for: Correction: How Etuaptmumk/Two-Eyed Seeing is used in Indigenous health research: A scoping review
Source: PLoS One. 2026 May 13;21(5):e0349369. doi: 10.1371/journal.pone.0349369 (PMC13170871; doi:10.1371/journal.pone.0349369)
Supplement: S2 File — (PDF) [file pone.0349369.s002.pdf]

PLOS ONE

RESEARCH ARTICLE

How *Etuaptmumk*/Two-Eyed Seeing is used in Indigenous health research: A scoping review

Sophie I. G. Roher[1,2*], **Debbie H. Martin**[3‡], **Ziwa Yu**[4], **Tara Pride**[5], **Marni Amirault**[3], **Jenny R. Rand**[3], **Anita C. Benoit**[6,7,8‡],

1 Institute for Circumpolar Health Research, Yellowknife, Northwest Territories, Canada, **2** Department of Anthropology, University of Toronto Mississauga, Toronto, Ontario, Canada, **3** Faculty of Health, Health Promotion Division, Dalhousie University, Halifax, Nova Scotia, Canada, **4** Pediatric ICU, BC Children's Hospital, Vancouver, British Columbia, Canada, **5** Faculty of Health Sciences, School of Occupational Therapy, Westerns3 University, London, Ontario, Canada, **6** Department of Health and Society, University of Toronto Scarborough, Toronto, Ontario, Canada, **7** Dalla Lana School of Public Health, University of Toronto, Toronto, Ontario, Canada, **8** Women's College Research Institute-Women's College Hospital, University of Toronto, Toronto, Ontario, Canada

‡ DHM and ACB are joint senior authors and contributed equally to this work.
* sophie.roher@mail.utoronto.ca

Abstract

Our scoping review sought to describe how *Etuaptmumk* or Two-Eyed Seeing is used and reported on in Indigenous health research. Using the JBI scoping review methodology, we extracted uses of *Etuaptmumk*/Two-Eyed Seeing from 83 articles and then categorized the reported uses of *Etuaptmumk*/Two-Eyed Seeing according to Huria et al.'s eight CONSIDER statement domains (governance, prioritization, relationships, methodologies, participation, capacity, analysis and interpretation, and dissemination). We found that while authors used *Etuaptmumk*/Two-Eyed Seeing in varied ways and at different stages of their research projects, characterizations of the guiding principle were often insufficiently described or overly simplified. This scoping review intends to contribute to a greater dialogue about how *Etuaptmumk*/Two-Eyed Seeing is conceptualized and used in Indigenous health research with the goal of encouraging more intentional reporting of the guiding principle.

OPEN ACCESS

Citation: Roher SIG, Martin DH, Yu Z, Pride T, Amirault M, Rand JR, et al. (2024) How *Etuaptmumk*/Two-Eyed Seeing is used in Indigenous health research: A scoping review. PLoS One 19(9): e0310247. https://doi.org/10.1371/journal.pone.0310247

Editor: Julia Morgan, University of Greenwich, UNITED KINGDOM OF GREAT BRITAIN AND NORTHERN IRELAND

Received: March 6, 2024

Accepted: August 23, 2024

Published: September 19, 2024

Copyright: © 2024 Roher et al. This is an open access article distributed under the terms of the Creative Commons Attribution License, which permits unrestricted use, distribution, and reproduction in any medium, provided the original author and source are credited.

Data availability statement: All relevant data are within the manuscript and its Supporting Information files.

Funding: Author SIGR is supported by a Canadian Institutes of Health Research (CIHR)'s Patient-Oriented Research Fellowship Award (grant # CIHR/170664; https://cihr-irsc.gc.ca/e…) and was previously supported by

Introduction

Etuaptmumk (E) is the Mi'kmaw word for Two-Eyed Seeing (TES), a guiding principle that has been increasingly taken up in Indigenous health research [1]. E/TES was first introduced to the academic community by Mi'kmaq Elders Drs. Albert and Murdena Marshall from Eskasoni First Nation and Dr. Cheryl Bartlett to guide the Integrative Science program at Unama'ki College [2,3]. Elders Drs. Albert and Murdena Marshall and Dr. Cheryl Bartlett (i.e., the original authors) described E/TES as: "learning to see from one eye with the strengths of Indigenous knowledges and ways of knowing, and from the other eye with the strengths of Western knowledges and ways of knowing… and learning to use both these eyes together, for the benefit of all" [4]. While E/TES has been used in academic research, Elder Dr. Albert Marshall explains that E/TES cannot be easily classified in academic terms. According to Elder

a CIHR Doctoral Research Award through the CIHR Institute of Aboriginal People's Health (grant # CIHR/152258; https://cihr-irsc.gc.ca/e/8668.html). DHM is supported by a Tier II Canada Research Chair in Indigenous Peoples' Health and Well-Being through the Canada Research Chairs Program (grant # CRC-2021-00436; https://www.chairs-chaires.gc.ca/home -accueil-eng.aspx). ACB was previously supported by an OHTN-CIHR New Investigator Award through CIHR (grant #APP268899; https://cihr-irsc.gc.ca/e/ 44181.html). TP was supported by a SSRHC Doctoral Fellowship and the Atlantic Indigenous Mentorship Kausattumi Grants Program Doctoral Award. JRR is supported through DHM's Canada Research Chair Program (grant # CRC-2021-00436; https://www.chairs-chaires.gc.ca/home -accueil-eng.aspx). The funders had no role in the study design, data collection and analysis, decision to publish, or preparation of the manuscript.

Competing interests: The authors have declared that no competing interests exist.

Dr. Albert Marshall, E/TES is a broader and more dynamic guiding principle for how to live one's life. It is a way of thinking and living that is deeply connected to spirit [5].

Since E/TES cannot be easily labeled using academic terms, it is fitting that there is no checkbox of things one needs to do to enact E/TES and little practical guidance is given to researchers for how to use E/TES in research. Indeed, we would argue that one of the strengths of E/TES is that it is fluid and dependent on contextual factors such as time, place, and people(s) involved. When E/TES is communicated by its original authors, it is often communicated orally [6]. Nevertheless, given that there is no practical guidance for how to use the guiding principle in research, and as E/TES is increasingly used in Indigenous health scholarship, it is also at greater risk of being misused or misrepresented [1,7,8]. Elder Dr. Albert Marshall noted: "The work can all too easily slip into a lazy, tokenistic approach in which E/TES and similar efforts quickly become mere jargon, trivialized, romanticized, co-opted, or used as a 'mechanism'" [7]. Since future academic work builds on previous academic work, it is important for researchers to clearly articulate how they are conceptualizing and using E/TES in their research projects so that we can develop a rich and nuanced understanding of the guiding principle.

Huria et al. developed the **CONS**ol**ID**ated crit**ER**ria (CONSIDER) statement for strengthening reporting of health research involving Indigenous Peoples and knowledges [9]. The CONSIDER statement consists of eight research domains that researchers should be attentive to when reporting about Indigenous engagement and the use of Indigenous knowledges in health research: governance, prioritization, relationships, methodologies, participation, capacity, analysis and interpretation, and dissemination.

To encourage more thoughtful engagement with E/TES, we carried out a scoping review to better understand how E/TES is used in Indigenous health research literature. Our review is intended to build on a previous scoping review, which found that E/TES is characterized and described in Indigenous health research in a variety of ways [1]. Our previous review identified seven ways that E/TES was *described* by the original authors and new authors. E/TES was characterized as a guide for life; co-learning journey; responsibility for future generations; numerous or diverse perspectives; spirit; decolonization and self-determination; and humans as part of the environment [1]. Since our goal is to strengthen the way that researchers are reporting their use of E/TES, in this scoping review we sought to examine how E/TES is *used* in Indigenous health research and to assess researchers' reported *usage* of E/TES through the CONSIDER statement's eight domains. Our intention is not to claim that there is 'one' 'right' way to use E/TES, nor are we claiming that we hold some sort of authority about how E/TES 'should' be used. Rather, our aim is to encourage all of us to be more thoughtful in the ways that we engage with and describe E/TES.

Methods

Our scoping review followed the methods outlined in the JBI Manual for Evidence Synthesis [10] (See S1 Protocol). We worked with the five stages of the York Framework, including having a team approach of regular and consistent meetings throughout the entire review process such as data extraction, analysis, and presentation [11] with clarifications as described by Levac et al. 2010 [12]. As recommended by the JBI scoping review guidelines, we used open coding, which included allocating the use of E/TES into overall categories, and we used a deductive approach, which involved mapping our data to the CONSIDER statement domains, which was our coding framework (we describe our process in greater detail below).

Our research question was: How is Etuaptmumk/Two-Eyed Seeing reportedly being used in Indigenous health research? The Preferred Reporting Items for Systematic Reviews and

Meta-analyses extension for Scoping Reviews (PRISMA-ScR) [13] guided the conduct and reporting of this scoping review, which is congruent with the JBI approach.

Eligibility criteria

The key concept underpinning our scoping review is 'Two-Eyed Seeing' known in the Mi'kmaw language as 'Etuaptmumk'. The main populations of interest were First Nations, Inuit, and Métis in Canada, though we also considered international scholarship using E/TES. Literature that involved non-Indigenous populations (e.g., non-Indigenous health professionals, educators, policy makers, and researchers) were included so long as the research aim aligned with CIHR's definition of Indigenous health research, which can be defined by "any field or discipline related to health and/or wellness that is conducted by, grounded in, or engaged with, First Nations, Inuit or Métis communities, societies or individuals and their wisdom, cultures, experiences or knowledge systems, as expressed in their dynamic forms, past and present" [14]. We considered scholarship across a variety of academic disciplines including health sciences, education, and biology, among others.

We included English-language literature published from Jan 1, 2004 (i.e., the year when E/TES was introduced to academia through the Integrative Science research program by Elders Drs. Albert and Murdena Marshall and Dr. Cheryl Bartlett [15]) until August 31, 2023. In terms of types of literature, we considered primary research of all study designs and reviews such as scoping or systematic reviews. Only abstracts that have a corresponding full-text publication were included.

Data sources

The following databases were searched by a JBI-trained health sciences librarian: Medline (PubMed) and Medline (Ovid), Academic Search Premier (EBSCOhost), PsycINFO (EBSCOhost), CINAHL (EBSCOhost), Bibliography of Indigenous Peoples in North America (EBSCOhost), and EMBASE (Ovid). We also searched for our key terms in the grey literature: the Indigenous Studies Portal (iPortal) and the Institute for Integrative Science & Health (IISH) website. Four key journals (International Journal of Indigenous Health, International Journal of Circumpolar Health, International Indigenous Policy Journal, and Pimatisiwin: A Journal of Aboriginal and Indigenous Community Health) were also searched. The search end date was August 31, 2023 with a search start date of January 1, 2004, aligning with our inclusion criteria.

Search strategy

Two key terms, 'Two-Eyed Seeing' or 'Etuaptmumk', were used to search the title, abstracts, and keywords for our databases with limits to English language sources. We searched our key terms one at a time using them as keywords as there are no synonyms or MeSH terms and then combining our individual searches using the Boolean operator "OR". The grey literature and four key journals were hand searched for relevancy using our two key terms. Reference lists of included literature were scanned for additional records. Finally, we contacted authors of conference abstracts to request potential full-text publications.

Citation management and screening process

Search results were imported into the online systematic review platform Covidence (Veritas, Melbourne, Australia) for screening. First, each title and abstract were independently assessed by two reviewers against pre-defined eligibility criteria. Potentially relevant citations were then

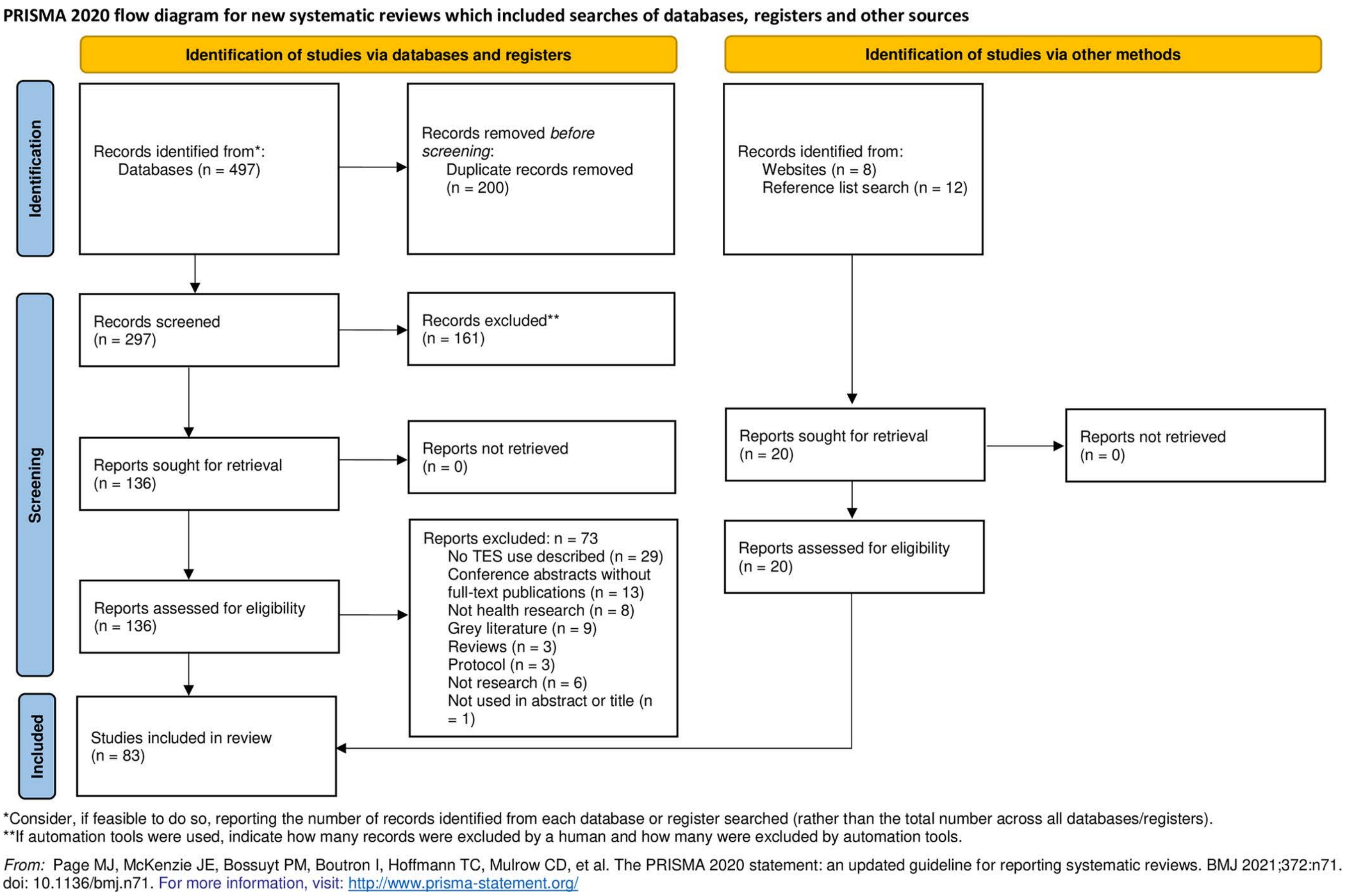

Fig 1. PRISMA-ScR flow diagram. Study selection process.

https://doi.org/10.1371/journal.pone.0310247.g001

retrieved in full-text and assessed again by two reviewers independently. To exclude a full-text article, reviewers needed to provide a rationale, which we summarized in Fig 1. Any disagreements that arose between reviewers were settled through discussion or by a third reviewer.

Data charting and synthesis

We extracted the data in two parts. First, a charting table in Microsoft Excel was created by authors to extract the following details from included literature: author(s), year of publication, research design, study objectives, research participants, and study location. The charting table was first trialed independently by each member of the review team on four full-text articles to ensure all relevant information was captured and that there was a mutual understanding of all data extraction terms. The remaining data extraction was completed with two members of the review team independently reviewing each article. Any disagreements were resolved through discussion or with a third reviewer, if required.

Second, we created a chart to extract authors' explicit descriptions of how E/TES was used to guide their research activities. In the chart, we categorized E/TES usage according to the eight domains from the CONSIDER statement [9]. To start, two members of the review team independently reviewed each article and extracted quotations that described how authors used E/TES in their research projects. When needed, disagreements were resolved through discussion or with a third reviewer. The quotations were then categorized according to the eight

research domains of the CONSIDER statement. The entire review team met multiple times to discuss the categorizations until consensus was reached. Finally, based on the categorizations, the review team worked together to create short summaries for each domain to describe how researchers were reporting their use of E/TES (see 'Domains describing the reported use of *Etuaptmumk*/Two-Eyed Seeing').

Results

The literature screening process is summarized in a PRISMA-ScR diagram (Fig 1). The search of databases resulted in 497 records. An additional 20 articles were included based on searching key websites, journals, and reviewing relevant articles' reference lists. After removing duplicates, 297 remained for title and abstract screening of our key terms, of which 136 progressed to full text review. Ultimately, 83 articles met the eligibility criteria and were included in our review.

Characteristics of included articles

An overall description of the included literature is presented in S1. All 83 articles were published after 2009 with 80 articles published since 2015. The included literature were journal articles (n = 82) and book chapters (n = 1). In terms of research design, 61 research articles employed an empirical design (i.e., research that is based in observation or measurement of phenomena and uses qualitative or quantitative methods to gather evidence [16]). Among these, 22 used community-based research design variations, such as community-based participatory research or participatory action research [17–38].

Domains describing the reported use of Etuaptmumk /Two-Eyed Seeing

The descriptions below illustrate how E/TES was used and characterized according to the CONSIDER statement. Some articles used E/TES according to more than one domain. Usage of E/TES was most often categorized in the CONSIDER statement's domains of methodologies (n = 46); relationships (n = 45); and analysis and interpretation (n = 36); followed by dissemination (n = 12) and capacity (n = 9); and was least often categorized in the domains of prioritization (n = 7); governance (n = 1); and participation (n = 1) (Fig 2).

To better demonstrate how E/TES was used and catalogued, we present Huria et al.'s definition of each CONSIDER statement domain, followed by a summary of how researchers' reported usage of E/TES aligned with that domain.

Domain 1: Relationships

According to Huria et al. [9], *relationships* consist of specified measures that "adhere and honour Indigenous ethical guidelines, processes, and approvals" for all Indigenous individuals or groups who may have different research approaches, reporting Indigenous involvement in the research process from funding to dissemination, and a description of the research team's expertise in Indigenous health and research.

The use of E/TES in relationships was mentioned in 45 articles. In seven articles, E/TES was described in relationships according to more than one of the characterizations in Huria et al.'s definition [29,32,37–41]. Fourteen articles described a role for E/TES in specific measures that adhere to and honour Indigenous ethical guidelines, processes, and approvals [17,27,32,33,37–40,42–47]. A role for E/TES in shaping Indigenous involvement in the research process was described in thirteen articles that focused on power dynamics, engagement throughout research phases, and forming the research team [29,32,34,37–39,41,48–53]; nine articles in the selection of

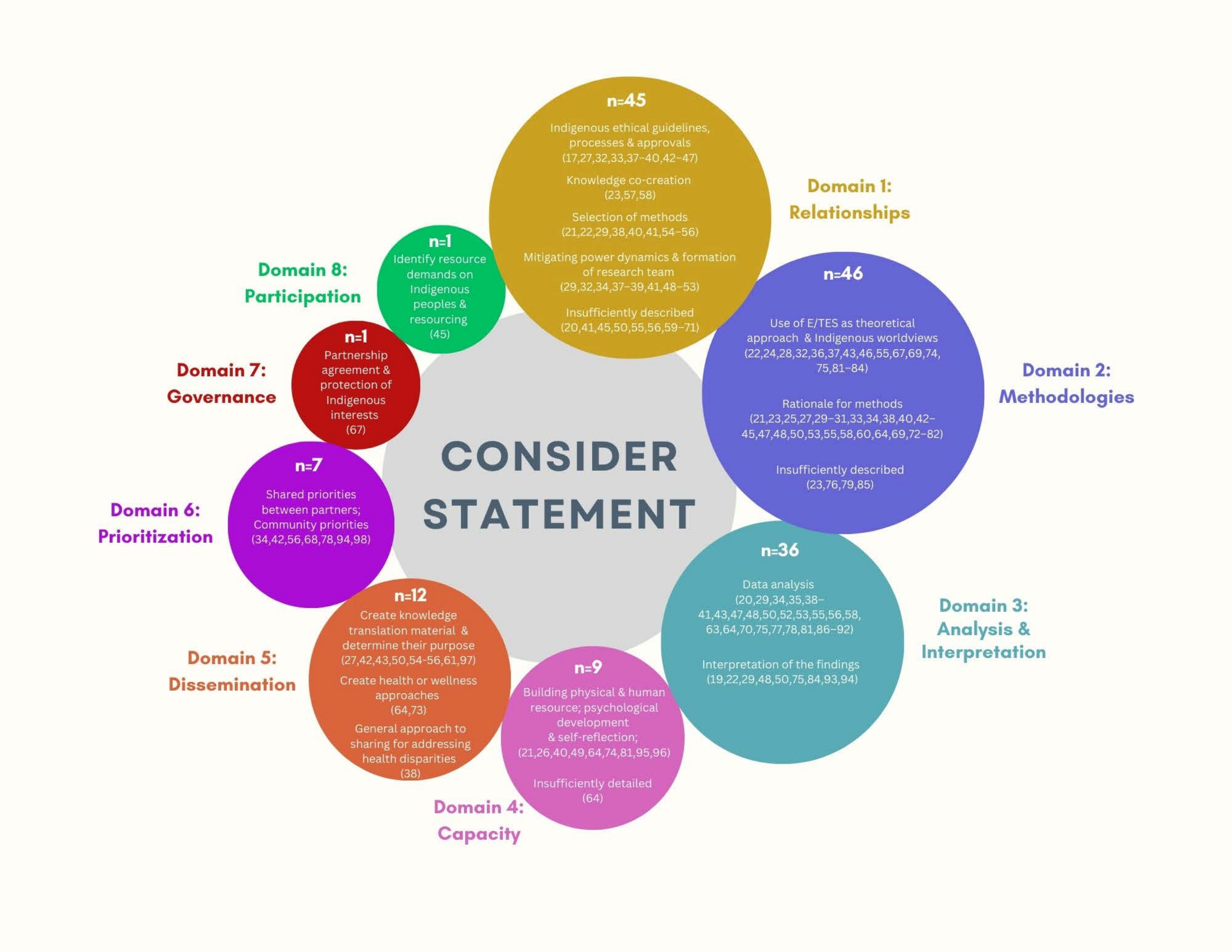

Fig 2. Schematic diagram. CONSIDER domains with specific components of the definitions reflected in the articles.

https://doi.org/10.1371/journal.pone.0310247.g002

methods [21,22,29,38,40,41,54–56]; and three articles for knowledge co-creation [23,57,58]. We discuss each of these articulations of E/TES in greater detail below.

Indigenous ethical guidelines, processes, and approvals. A role for E/TES in specific measures that adhere to and honour Indigenous ethical guidelines, processes and approvals was articulated in fourteen articles [17,27,32,33,37–40,42–47]. In five articles, E/TES was part of the more formal aspects of preparing for research to take place–including the establishment of guidelines for how to undertake the work [32,40,42,43,45]. In four articles, E/TES was used to build authentic relationships that facilitated a safe and trusting environment in which to share [33,38,40,44]. In two articles, E/TES was part of reviewing adaptations of existing programs to ensure cultural appropriateness and sensitivity [17,27]. In one article, E/TES was involved in processes for ensuring that the appropriate approvals were in place ahead of data collection [39], and in another, E/TES was reflected through ceremonies at the start of community engagement sessions to recognize the people and ancestors whose land the research was conducted upon [32]. Finally, in one article, an Elder's involvement in the research process was part of their E/TES approach [47].

Mitigating power dynamics and the formation of the research team. For some authors, E/TES included consciously considering power dynamics within a team [37,39,48,49]. For

instance, Mantyka-Pringle et al. [48] indicated that E/TES contributed a means for knowledge co-production that was 'power neutral' in the sense that multiple modalities of data capture were included, and that feedback on the process was elicited from various representatives of the research project, including local people, Elders, social and natural scientists, and government workers. Hall [49] indicated that although the funding was held at an academic institution (therefore creating an uneven power relationship at the outset), this was somewhat mitigated by having data collection led entirely by an Elder.

In other examples, authors described how E/TES was considered in relationships throughout different stages of the research process. In terms of data analysis, some authors indicated that this was undertaken specifically by both Indigenous and non-Indigenous peoples (i.e., a non-Indigenous researcher and Indigenous research assistant) [41], whereas in cases where the analysis was done by a team of non-Indigenous peoples, authors described re-engaging with the governing circle (which consisted of Indigenous peoples) to present the results for their feedback and comments [39].

For some researchers, E/TES shaped the composition of the research team [29,32,34,38,50–53]. For example, Wright et al. explained that in keeping with their E/TES approach, the study was collaboratively designed by a non-Indigenous nurse practitioner, a First Nations nurse and PhD student, a Métis scholar, and members of the Indigenous community in Hamilton [51]. Similarly, Rowan et al. [50] described how E/TES shaped the make-up of the study team in their scoping review process and they outlined the types of expertise brought to bear on the process by the various people involved. Both western and Indigenous thinkers were brought together in a shared space to exchange knowledge. These gatherings were important throughout the scoping review process, but particularly at the outset in shaping the research question.

Selection of methods. Some authors described involving Indigenous advisory committees, research participants, and research team members in decisions about the methods selected for the study [21,22,29,38,40,41,54–56]. Marsh [40] described that the involvement of an Indigenous advisory group, Elders, and the research committee ensured that the research process itself was culturally safe.

In one instance, authors specifically mentioned that relationships with the Indigenous community provided a means to verify the results of the research. "In Mi'kma'ki Two-Eyed Seeing collaborations are helping to create better and broader understandings of netukulimk within communities. The integrative approach is conducive to gathering Indigenous fisheries knowledge from active fishers and Elders participate in evidence verification" [56].

Knowledge co-creation. Another way that authors described their research relationships was through knowledge co-creation, whereby research teams indicated that Indigenous peoples were instrumental in forming a part of the research process itself [23,57,58]. Crooks et al. described this as both a collaborative and community-driven process that opened space to co-create research questions, identify appropriate methods, and ensure that data was being interpreted appropriately [23]. Similarly, Castleden et al. described the involvement of an advisory committee that consisted of Indigenous and non-Indigenous water experts that informed the research process from design to dissemination [57]. For Marsh et al. [58], co-creation involved the presence of an Elder during storytelling sessions which was intended to allow research participants to develop a connection to the spirit world through traditional teachings, and to offer a space for healing as they shared their own stories.

Insufficiently described. Notably, of the 45 articles, there were 19 articles whose usage of E/TES aligned generally with Huria et al.'s definition of 'relationships' but did not clearly describe what Indigenous involvement looked like in their projects and how this aligned with E/TES [20,41,45,50,55,56,59–71]. For example, McKivett [62] stated that they carried out E/TES by bringing together the Indigenous community, healthcare practitioners, medical

educators, and medical students as key research stakeholders in a way that centers Indigenous worldviews; nevertheless, it was unclear what this looked like in their study and how it aligned with their conceptualization of E/TES. Notably, of the 19 articles, we categorized five articles as both clearly and not clearly describing relationships because, in some sections of their articles they clearly described E/TES, while in other places they did not [41,45,50,55,56].

Domain 2: Methodologies

Methodologies consist of describing the methodological approach including the rationale for the methods used and implication of Indigenous people involved in the research, describing Indigenous worldviews as well as how the methodology considers the physical, social, economic and cultural environment of study participants [9].

The use of E/TES in methodologies was described in 46 articles. 17 articles reported using E/TES in their descriptions of Indigenous worldviews [22,24,28,32,36,37,43,46,55,67,69,72–77], and 35 articles reported using E/TES in the methodological approach including the rationale for the methods [21,23,25,27,29–31,33,34,38,40,42–45,47,48,50,53,55,58,60,64,69,72–75,78–84]. Five of the articles reported E/TES both as a rationale for the methods and in their descriptions of Indigenous worldviews [55,69,72,73,75]. The implication of Indigenous peoples involved in the research was not clearly described in relation to E/TES, nor were considerations for the physical, social, economic, and cultural environment of study participants.

Use of E/TES as theoretical approach. Some authors reported using E/TES as an overarching theory or approach for bringing together Indigenous and Western knowledges [22,24,28,32,36,37,43,46,55,67,69,72–77]. For example, Rand employed E/TES as a theoretical framework to bridge Inuit Quajimajatuqangit (IQ) and postcolonial theory [24]. Similarly, Snooks et al. used E/TES by "blending together…feminist, Indigenous, decolonial, and participatory action frameworks" [32]. Other authors used E/TES as a theoretical approach along with Indigenous or decolonizing methodologies. For instance, Quinn used E/TES with a Relational Worldview Model and decolonizing Indigenous research epistemologies to guide their study questions [46]. Similarly, Ward et al. used E/TES conceptually to recognize the partiality of Western knowledge and the fact that Western knowledges need to be located within Indigenous histories and worldviews [36]. Ward et al. described that E/TES encouraged them to reflect on and actively work to address power imbalances in the research process by collaborating with Indigenous peoples throughout and ensuring that the study benefits Indigenous peoples and communities who are taking part [36,37].

Rationale for methods. Some researchers conceptualized and used E/TES by including both Indigenous and Western methodologies, methods, or perspectives in their research [21,23,25,27,29–31,33,34,38,40,42–45,47,48,50,53,55,58,60,64,69,72–75,78–84]. This was referred to by some authors as a 'mixed methods' approach [75]. One example of this can be found in Rowan et al.'s scoping review which brought together Indigenous and Western perspectives in different ways at different points in the study [50]. According to Rowan et al., E/TES encouraged the researchers to form a balanced team of Western and Indigenous thinkers; to create a research question that recognized Indigenous knowledge and Western understandings of evidence; to use search term parameters that came from Indigenous perspectives; and to employ both Western and Indigenous criteria to label and extract data. Similarly, Poirier and Tait Neufeld used E/TES by employing both Indigenous sharing circles and Western reflexive thematic analysis in their research design [31].

While some researchers understood E/TES as a theory or approach for bringing together Indigenous and non-Indigenous knowledges, others conceptualized and used E/TES by centering Indigenous knowledges and methodologies [43,55,69,73]. For example, some

researchers employed E/TES by taking up methods that honour the oral tradition, such as interviews [43,55] and discussion groups [55]. Similarly, in Hall et al.'s study, E/TES enabled the researchers to use Indigenous methodologies such as storytelling and knowledge gardening [73]. According to Hall et al., E/TES enabled them to prioritize Indigenous governance and cultural guidance and ensured that Western timeframes did not dictate their research timelines. Additionally, E/TES meant that they used culturally-rooted analyses to validate their findings when Western research methods were used.

For some researchers, E/TES prompted them to 'Indigenize' western methodologies. For example, Carter et al. asserted that they conceptualized E/TES as a theory "for [how] Indigenous theory and non-Indigenous methods [could] be harmonized" [74] and used E/TES by Indigenizing their narrative methodology. For the authors, this meant that the non-Indigenous researcher "acknowledged with curiosity and respect the ways the participants interpreted experiences, which were outside of her worldview" and, in return, "participants helped [the researcher] understand by explaining the context and their beliefs" [74].

Insufficiently described. It is important to note that although some researchers reported that they used E/TES as a theoretical approach, methodology, or method, they did not always articulate how E/TES was used in their study [85]. For example, in Crook et al.'s article about a mental health first aid course, they described using E/TES as an approach to data collection and they outlined the ways they saw E/TES aligning with other Indigenous frameworks; however, they did not provide specific examples of what E/TES looked like in their study [23]. Similarly, Blangy et al. simply wrote that they adopted a 'Two-Eyed Seeing' framework without a greater explanation of how this looked in practice [80]. Stelkia et al. asserted that they embodied a E/TES approach, respecting distinct perspectives of diverse Nations in the region, however, they did not provide further detail [83].

Domain 3: Analysis and interpretation

Analysis and interpretation refers to specifying "how the research analysis and reporting supported critical inquiry and a strength-based approach inclusive of Indigenous values" [9].

E/TES was used in the analysis and interpretation sections of 36 articles. Among the 36 articles, 31 articles included E/TES in data analysis [20,29,34,35,38–41,43,47,48,50,52,53,55,56,58,63,64,70,73,74,81,82,86–92], and nine articles focused on using E/TES in the interpretation of findings [19,22,29,48,50,73,77,93,94]. Three articles were included in both analysis and interpretation [29,48,73].

Data analysis. Thirty-one articles included E/TES in data analysis [20,29,34,35,38–41,43,47,48,50,52,53,55,56,58,63,64,70,73,74,81,82,86–92]. E/TES often involved having Indigenous and non-Indigenous people involved in data analysis–either as a collaborative process with independent handling of the data [20,39,40,55,87], with the Indigenous perspective following the primary analysis to contextualize the data [41,70], or stating the inclusion of multiple perspectives in data analysis [29,34,35,38,43,50,52,53,56,63,64,74,81,82,86,88–92]. For example, Wright et al. [55,87,88] discussed engaging in a collaborative data analysis process by having an Indigenous research assistant work with a non-Indigenous researcher whereby the data was analyzed independently, then coming together to compare and contrast their analyses. This process required self-reflection and that both individuals carry with them their respective epistemologies and ontologies to the data analysis process with a particular focus on the non-Indigenous researcher taking note of the differences [55]. Another study involved a more iterative data analysis process with independent handling of the data by two Indigenous research team members who would then come to a consensus on the final coding which was reviewed by a non-Indigenous senior research team member to ensure consistency

and accuracy in the coding [39]. In this study, a team approach involving both Indigenous and non-Indigenous members was implemented to review the final codes [39]. In another study, Wright et al. discussed what is typically considered member-checking in qualitative research as an additional process to include Indigenous perspectives which would follow a primary analysis [41]. Data analysis was described as including multiple perspectives with limited details on how data analysis was practiced or explicitly stating where Indigenous perspectives were included [20,43,48,50,58,64,86]. Some articles reported that the inclusion of E/TES in data analysis allowed them to view the data through a strength-based or Indigenous lens [43,70,87], to capture cultural nuances or the impact of colonization on the topic of interest [43,86], or to enhance rigour of the findings [43,81]. The more detailed and iterative analyses often explicitly stated the importance of Indigenous epistemologies and ontologies [39,55].

Additionally, there were some articles that used E/TES by employing Indigenous and Western approaches to data analysis. For example, Roburn described utilizing both a Western social media analysis and Indigenous worldviews to analyze social media expressions [90]. Similarly, Sam et al. used E/TES by contextualizing data analysis as Indigenous storytelling to make sense of participants' experiences [82]. And finally, Vorobyova et al. used E/TES by analyzing perspectives of cultural safety amongst Indigenous and White older adults living with HIV [35].

Data interpretation. Nine articles focused on using E/TES in the interpretation of findings [19,22,29,48,50,73,77,93,94]. Some articles focused on bringing different perspectives together without explaining the influence of these perspectives on the data. For instance, one article simply stated that it allowed the authors to use Indigenous perspectives to explain findings [22]. Similarly, others used E/TES in a very broad sense to interpret or explain the relevance of the authors' work with regards to health, illness, healing, or treatment. E/TES was used to explain and understand illnesses and treatments characterized in a biomedical model and reflecting on the Indigenous perspectives or visualizations of illnesses and healing [19,50,77,94]. These articles did not give specific descriptions of what E/TES looked like in their analysis and interpretation.

In contrast, there were two articles that more specifically described their use of E/TES [48,93]. For example, one author described a role for E/TES in interpreting participants' responses about the ways they adapt to changes brought on by colonialism and their appreciation of aspects of contemporary life [93], which aligns with E/TES's understanding that perspectives are always changing. Another author reflected on the tensions of trying to reconcile diverse knowledge systems [48].

Domain 4: Capacity

Capacity involves explaining "how the research supported the development and maintenance of Indigenous research capacity" and "how the research team undertook professional development opportunities to develop the capacity to partner with Indigenous stakeholders" [9].

The use of E/TES was consistent with Huria et al.'s definition of capacity in nine papers [21,26,40,49,64,72,74,95,96]. Some authors described their use of E/TES in their research in relation to building physical and human resource capacity (i.e., training Indigenous facilitators [40] and delivering a baccalaureate-level program in and for the community [95]), while others (n = 6) explained their use of E/TES in terms of psychological development and self-reflection. That is, authors saw E/TES as enabling researcher(s) to engage in greater reflection about their personal beliefs and values [96], consider different worldviews and understand the importance of Indigenous culture [21,26,64], build capacity to do culturally-rooted research [49], and promote dialogue and discussion between people of different worldviews [72]. Additionally, some

understood E/TES as encouraging self-reflection by the non-Indigenous researchers and ensuring that the non-Indigenous researchers were cognizant of Indigenous perspectives [74].

As with articles in other domains, some authors did not sufficiently detail how E/TES was used in relation to capacity building. Whiting described distinct worlds coming together through Indigenous and mainstream service providers as gateways to bring the best aspects of these worlds to meet client and community needs [64]. We classified this example under 'capacity' because we understood this as aligning with the concept of co-learning between cultural and mainstream healthcare providers. However, details were not provided on how coming together was done or how the best of both worlds was chosen.

Domain 5: Dissemination

Dissemination involves a description of "the dissemination of the research findings to relevant Indigenous governing bodies and peoples" and of "the process for knowledge translation and implementation to support Indigenous advancement" [9].

The use of E/TES was described in dissemination activities in 12 articles [27,38,42,43,50,54–56,61,64,79,97]. All but one article [42] failed to explicitly acknowledge to whom the findings were being shared. The procedural aspects of dissemination and how the knowledge could be used to support Indigenous advancement was also absent. Among the 12 articles, there were two areas of focus on the use of Indigenous and non-Indigenous perspectives which were to create knowledge translation materials [27,42,43,50,54–56,61,97], and health or wellness approaches [64,79]. One article described knowledge sharing more generally as meaningfully sharing and applying the research to address community health disparities [38].

Indigenous and non-Indigenous perspectives were brought together in 9 articles to inform the type of knowledge translation materials created (e.g., short stories, academic publications, teaching tools, instrument development, fact sheets, communication and management strategies, consensus statement) [27,42,50,61,97] or to establish the purpose of the materials (e.g., health promotion, health literacy, and awareness messages; greater knowledge on research topics and Indigenous roles; explain how Indigenous worldviews inform the research process; inform policymaking and programming) [27,42,43,54,56]. Two articles focused on co-learning as part of dissemination [42,56]. Three articles stated the role of E/TES in knowledge exchange and influencing future knowledge translation efforts [43,55,56].

The use of Indigenous and non-Indigenous perspectives to create health or wellness approaches was described in two articles [64,79]. However, details were not provided on how this was done or how aspects of each perspective were chosen.

Also, all articles described a role for E/TES in advancing Indigenous health or knowledge, but they did not describe how E/TES "support[ed] Indigenous advancement" [9] with regard to research capacity, policy, and investment specifically within the context of dissemination.

Domain 6: Prioritization

Prioritization refers to how the research aims stem from the identified priorities of Indigenous individuals or groups and empirical evidence [9].

The use of E/TES in prioritization was characterized in seven papers [34,42,56,68,82,94,98]. These papers describe "shared priorities" between research partners [94], how the research team came together [82], for example "through a shared topic of interest" [68], or how research can be a means to assert worldviews that are more congruent, representative, and therefore meaningful to Indigenous peoples [42]. VanEvery et al. described that in keeping with a E/TES approach, they carried out a pre-study community engagement session with researchers, healthcare providers Elders, youth, a nurse research coordinator, and a privacy

officer to ensure the study goals reflected community priorities [34]. Though not specific to research per se, Clark emphasized the importance of using E/TES in health records for Manitoba Inuit by incorporating Inuit perspectives and traditional knowledge [98]. One clear example is evidenced from a paper by McMillan and Prosper. They report on how the Atlantic Policy Congress of First Nations Chiefs were ascertaining how fisheries scientists can best engage with Indigenous communities. Their policy analysis team worked in collaboration with Fisheries and Oceans Canada, particularly the Aboriginal Aquatic Resource and Oceans Management program, to embark on a co-learning journey with fisheries scientists to generate sustainable interface with Indigenous fishers, foster reciprocal relations, and create a management plan that reflects values that are important to the Mi'kmaw Nation [56].

Domain 7: Governance

Governance is characterized by "partnership agreements between research institutions with Indigenous-governing organizations", having accountability and review mechanisms within agreements that also addresses how to minimize harms, and the specification on how agreements protect Indigenous intellectual property and knowledge stemming from research including financial and intellectual benefits [9].

Usage of E/TES in governance (e.g., research agreements, memorandums of understandings) as described by Huria et al. was reflected in one article. Although several papers mention the importance of governance, or in some cases, describe governing bodies that oversaw the research, the definition as it is conceived by Huria et al. requires more specificity than this. The definition asks for evidence that there are partnership agreements in place, and for a description of how those agreements aim to protect the interests of Indigenous Peoples. In the single example that we identified, Denny and Fanning noted that "given the parties involved, any co-management approach being discussed must acknowledge and recognize the value of multiple realities. Pluralism in this case refers to the multiple realities related to views on allocation and access, provincial and customary laws, traditional knowledge and science, values and economics, and natural and legal relationships to salmon, all of which can offer depth and insight in the design of co-management" [67]. Although this example does not specifically identify *research* governance, we felt it was important to include as it strives to articulate principles and mechanisms through which co-management can take place–which, although focused in this case on salmon, could apply to research more generally.

Domain 8: Participation

According to Huria et al., *participation* consists of specifying how individual and collective consent was obtained for future research on data or samples collected, how data and biological samples were stored, explaining the processes of removal from traditional lands, if done, and of disposal; how resource demands on Indigenous participants and communities were identified and agreed upon including any resourcing for participation, knowledge, and expertise [9].

Although 83 articles were eligible for our review, only one article met the criteria within the participation domain which was related to how resource demands on Indigenous participants and communities were identified and agreed upon including any resourcing for participation, knowledge, and expertise [9]. Hatala et al. described providing ceremonial and western-based honorariums during data collection as well as research meetings to demonstrate respect and acknowledge the contributions of their study participants [45].

Discussion

The motivation for this scoping review came from Elder Dr. Albert Marshall's concern that as E/TES is increasingly used in research, it is at greater risk of being tokenized [7]. Our goal was

to push ourselves and others to do better. We thought that if we could gain a greater understanding of how E/TES is used in Indigenous health research, then we could help to support ourselves and others to use E/TES in more nuanced and thoughtful ways. Additionally, since the CONSIDER statement offers clear and specific reporting standards for research with Indigenous Peoples, we decided to evaluate the use of E/TES according to the CONSIDER statement's eight research domains. It is important to note that the goal of this article is not to criticize individual authors for their descriptions, but rather to strive for more conscientious and intentional collective reporting of E/TES. Additionally, we are not trying to provide a prescriptive way of how to use or report on E/TES. Instead, we are hoping to encourage researchers to think critically and intentionally about the ways they are using E/TES in their respective studies and to clearly articulate this in their reports, publications, and presentations.

With these objectives in mind, rather than go through each CONSIDER domain one-by-one, in this discussion, we instead reflect on cross-cutting themes to provoke critical thinking about the ways that E/TES is used in Indigenous health research. The discussion is made up of two parts. First, we reflect on overarching themes from our scoping review and second, we consider the strengths and limitations of using the CONSIDER statement.

Part 1: Reflections on key themes

E/TES was used in varied ways. One central theme in our scoping review was that E/TES was used in a variety of innovative and unique ways and that its use depended on each study's specific context. In some articles, E/TES informed the topic under study, the people(s) involved, and the way(s) the project was governed. In other studies, E/TES shaped how data was collected, analyzed, and/or shared. The diversity of uses of E/TES demonstrate the adaptability and resonance of the guiding principle. As we mentioned in the introduction, one of the strengths of E/TES is that it can be used in multi-dimensional ways.

While the studies in this scoping review collectively covered all eight domains of the CONSIDER statement, no single article captured all eight of the domains as they were articulated by Huria et al. There remains much room for expansion on the use of E/TES, particularly since its use was less frequently reported on in research activities that might contribute to greater Indigenous research sovereignty and governance (i.e., the domains of governance, participation, etc.). Indeed, it is possible that E/TES is less commonly used in these domains because they are predicated on the creation of authentic and meaningful research relationships and necessitate a level of specificity, commitment, and institutional support, which require more time and resources and take longer to establish.

While E/TES was minimally used in the domains of governance and participation, it was perhaps unsurprising that E/TES was used most in the domains of 'methodologies' (n = 46), and 'relationships' (n = 45), 'analysis and interpretation' (n = 36) because there has been quite a bit of scholarship written about Indigenous engagement, community-based research, and Indigenous methodologies [99–101]. E/TES may align with the CONSIDER statement's definition of 'relationships' because the guiding principle is relational; it honours the interconnectedness of human beings and the environment and encourages co-learning between different people and perspectives [1,4,7]. Similarly, E/TES may more frequently align with Huria et al.'s definitions of 'methodologies' and 'analysis and interpretation' because E/TES is often articulated as a way of thinking about knowledge and knowledge creation–and particularly, as a way of thinking about how to bring Indigenous and non-Indigenous knowledges together. Since researchers often try to define what knowledge is and how it is generated when they are deciding which methodologies and strategies for analysis and interpretation they are going to use in their projects, there is great alignment between E/TES and these domains.

Nonetheless, even though many researchers used E/TES to inform their methods, Elder Dr. Albert Marshall and Dr. Cheryl Bartlett have been clear that E/TES is not a methodology or method [1]; it is a guiding principle for life and much broader than research [1]. As a result, we must not limit our conceptualizations of E/TES by using it solely as a methodology, method, or framework for analysis and interpretation. Rather, we must continually push the limits of our thinking beyond categorical descriptions and towards, what Elders Drs. Albert and Murdena Marshall and Dr. Bartlett describe as *integrative* thinking; actively pursuing the unique linkages, connections, and interpretations that emerge when diverse understandings are brought together. It is important to continue to think about and use E/TES broadly, aiming for it to be incorporated into each of the domains identified in the CONSIDER statement, and to also reflect on what synergies emerge across domains when E/TES is used to guide the work.

E/TES was insufficiently described. Another key theme across the domains was that the use of E/TES was often not well described. Authors commonly reported *that* they used E/TES or they discussed the general benefits of E/TES; however, they did not clearly articulate *how* they used E/TES. This made it challenging at times to categorize uses of E/TES according to the CONSIDER statement. The lack of sufficient descriptions of E/TES aligns with a finding from our previous scoping review where we observed that authors extensively used direct quotes from the original authors to define and characterize E/TES and frequently would not use their own words to describe E/TES [1]. It is possible that researchers are not clearly describing E/TES and how they used it in their studies because they do not feel comfortable with E/TES, or because they are concerned that they are not using E/TES in the 'right' way. Alternatively, it may be that researchers believe that E/TES can simply be reduced to using Indigenous perspectives through methods and the inclusion of Indigenous people alongside non-Indigenous perspectives without meaningful consideration to what Indigenous ways of knowing and doing might mean for Indigenous communities or Peoples that goes beyond the research. Simply collecting information or data from Indigenous people does not mean that the wisdom of local or shared cultural understandings pertaining to Indigenous knowledges has been respectfully gathered [102].

Relatedly, we observed that E/TES seemed to be commonly used by non-Indigenous researchers [55,66,70,74,87]. This may be because E/TES is considered a more 'comfortable' principle for non-Indigenous researchers than perhaps some other Indigenous principles. For example, Wright et al. asserted that they used E/TES rather than an Indigenous theory because the researcher was not Indigenous and thus felt that she could not appropriately conduct a study grounded in Indigenous knowledges [55]. It is possible that descriptions of E/TES usage are insufficiently described because researchers may be hesitant to go deeper with the guiding principle and recognize the multiple facets of E/TES, (i.e., the importance of spirit, co-learning, and the desire to work for seven generations into the future). Non-Indigenous researchers who choose E/TES over what they might consider 'Indigenous theory(ies)' for comfort should consider the multiple facets of E/TES. Additionally, they might want to reflect and ask themselves why they are comfortable and whether they are simply being complacent. Using an Indigenous guiding principle means that Indigenous knowledges should be centred and valued; this is not to say that other knowledges should be rejected if valued and requested by those for whom the research is intended to benefit.

The fact that some researchers are insufficiently describing E/TES speaks directly to Elder Dr. Albert Marshall's concern that E/TES can become tokenized or watered down if individuals are not intentional about the ways that they are using and reporting their usage of the guiding principle. This theme underscores the need for researchers to clearly articulate E/TES and its use in research.

E/TES was oversimplified. Another theme we noticed across categories was that some uses of E/TES were oversimplified in the ways they were described. For example, in Victor et al.'s article, the authors reported that bringing Indigenous and non-Indigenous peoples together "led organically" to a E/TES practice [70]. This article seemed to suggest that simply having Indigenous and non-Indigenous people involved in a project meant that E/TES was necessarily taking place. In our previous scoping review, we identified seven descriptions of E/TES that were used by the original authors and new authors. E/TES was described as: a guide for life; co-learning journey; responsibility for future generations; numerous or diverse perspectives; spirit; decolonization and self-determination; and humans as part of the environment [1]. When authors oversimplify E/TES by suggesting that it can happen passively by simply bringing Indigenous and non-Indigenous peoples together, it misses the expansiveness, richness, and active nature of the guiding principle. E/TES is not a passive outcome; instead, it requires an engaged and deliberate process of co-learning, dialogue, and knowledge gardening. We intentionally bring forward the term 'knowledge gardening' here, as it is a term often used within the context of E/TES to describe the process by which we come to understand that we have options and alternatives as to how we participate in the web of life (e.g., the fostering of a new consciousness). Elder Dr. Albert Marshall eloquently explains that "seeds only germinate when environmental conditions are appropriate" [103], which he further elaborates to mean that sometimes a seed or idea (i.e., knowledge) may be planted, but it will take the right conditions, nourishment, and patience for it to begin to germinate and grow.

Clear description of E/TE. Finally, we believe that it is important to emphasize that there were some authors who clearly and explicitly articulated how they used E/TES in their studies. These examples illustrate that clear articulations of E/TES are possible. For example, Rowan et al. reported using E/TES differently at different stages in their research project [50]. At the start of the project, E/TES exposed the researchers to different ways of knowing and encouraged them to include cultural activities and ceremony. E/TES also prompted the research team to have a balance of Western and Indigenous thinkers who were open to different ways of knowing. As the project continued, the researchers' use of E/TES changed. E/TES informed the research question by bringing together Indigenous knowledges with Western understandings of quality of evidence; it encouraged the team to use both Indigenous-led perspectives and Western-based tools to search and screen the literature; and it prompted the researchers to switch from a systematic review to a scoping review so that the study was more open to Indigenous knowledges and contexts. Rowan et al.'s in-depth and intentional descriptions demonstrate that E/TES can offer different strengths, opportunities, and ways of thinking at different points in a project. This is not to say that there is one *right* way to use or report E/TES, but rather to say that what is 'right' about this description is that the authors were clear and considerate about how they used and reported their usage of E/TES.

Part 2: Strengths and limits of the CONSIDER statement

This was the first study that employed the CONSIDER statement to evaluate uses of E/TES. Ultimately, we found that the CONSIDER statement offered a valuable framework through which to assess how researchers were reportedly using E/TES. One of the strengths of the CONSIDER statement is that the definitions for each domain focus on practical activities and outcomes rather than on theoretical ideas. For example, 'governance' does not speak to research governance in general but about specific partnership agreements. Similarly, 'relationships' is not about an overarching partnership approach, but instead *how* Indigenous peoples

and communities were involved in all stages of the research. As a result, the CONSIDER statement encourages researchers to move away from buzz words and to instead be clear about the specific ways they enacted E/TES. The clarity demanded in the CONSIDER statement was important because it drew our attention to situations where E/TES was and was not clearly described and thus deepened our understanding of how E/TES is being reported.

While Huria et al.'s CONSIDER domains provided a useful way of analyzing researchers' reported use of E/TES, there were key pieces that were missing from the CONSIDER statement. For example, our analysis helped to illuminate that spirit and connection to land are not included as part of the domains. For example, Hall et al. wrote that part of their use of E/TES included "recognizing that consciousness exists between human and more-than-human environments, and the need to bring knowledge together in timely, patient, and respectful ways" [73]. We found that it was hard to categorize this use of E/TES in the CONSIDER statement because spirit and connection to land, which were key elements to their use of E/TES, are not part of the CONSIDER definitions. This finding suggests that there may be an opportunity to expand the CONSIDER statement definitions and domains to include spirit and connection to all beings.

Relatedly, and perhaps connected to this absence of spirit and connection to land, is the fundamental importance of Indigenous worldview as informing elements of all domains. The CONSIDER statement currently includes Indigenous worldviews within the 'methodologies' domain, which we would argue is problematic. Many decades of Indigenous scholarship have highlighted Indigenous worldviews as synonymous with Indigenous ways of knowing, which moves far beyond the idea of methodology [99–101].

Study limitations

There were some limitations to our study. We were interested in direct and explicit descriptions of how E/TES was used. As a result, if an article used CBPR and E/TES but discussed Indigenous engagement only in relation to CBPR and not E/TES, then we did not include it in our extraction. Similarly, we found that E/TES was less commonly applied in the domains of governance and prioritization. The absence of these domains does not mean that they were not part of the research process; rather, it means that they were not reported in relation to E/TES.

There was one article that did not fit into the CONSIDER statement's domains because the authors did not provide sufficient detail about how E/TES was used in their study. Gray et al. wrote: "This study is also an example of how western science and Indigenous knowledge can be merged for the benefit of both knowledge systems in what is known to Indigenous groups as etuaptmumk or 'two-eyed seeing'" [104]. Based on this description, it was difficult to determine how this study used or was an example of E/TES despite meeting our inclusion criteria.

Since we only included explicit usage of E/TES when we extracted information from the project, we may have missed some elements about how E/TES was used. This issue speaks to the need for researchers to be attentive to how they are describing their use of E/TES. Additionally, we want to be clear that the responsibility for better reporting of E/TES does not entirely lie with researchers. Since researchers are bound by the reporting requirements and limitations of the journals where they publish (e.g., low word counts and strict reporting structures), it is imperative for journals to also take steps to support better reporting.

In fact, we would argue that it may be increasingly important to publish research protocols related to Indigenous health research because the protocols can give both academic and community researchers the opportunities to expand on the research process and reflect more deeply on the process of doing research. It may also serve as an important mentoring tool for early career Indigenous researchers and shape the approaches undertaken by non-Indigenous

researchers. Conducting Indigenous health research frequently requires significant and ongoing relationship building that occurs over an extended period, but is rarely documented to share lessons learned. Also, for many Indigenous and allied researchers, relationship building does not occur only upon receiving funding. A true commitment to community is made prior to asking Indigenous peoples to consult, participate, collaborate, or preferentially co-lead or lead research depending on their needs. As a result, sharing the process of establishing Indigenous governance, sovereignty, and self-determination throughout the research process can support the community in developing sustainable research frameworks as well as obtaining increased independence in pursuing research priorities.

In our paper, E/TES usage was largely found in relation to research conducted in Canada from coast to coast including the Territories with First Nations, Inuit, and Métis Peoples, showing its adaptability to various cultures and research contexts. Nevertheless, an important limitation of our paper would be to consider how and whether Indigenous Peoples from across the globe would be able to conceptualize their research process through E/TES, which may entail sharing some of the same values of the guiding principle [1] or even agreeing with the notion that bringing Indigenous perspectives and non-Indigenous perspectives together can be beneficial in research with and for Indigenous Peoples. We contend that Indigenous Peoples from regions outside of Canada have thought processes that are similar to E/TES in terms of bringing together diverse perspectives which includes local Indigenous perspectives. These are worth future explorations given the tensions that may exist between Indigenous and non-Indigenous worldviews within academia that sustain power differentials in terms of who teaches and who is taught as well as who researches and who is researched. For example, within education, *Strong like Two People* stemmed from the words of Tlicho Chief Jimmy Bruneau to accept "…the worldview of the white man but to never lose the skills, teachings and traditions of the Dene" [105]. Similarly, for educational purposes, the *Alaska Rural Systemic Initiative* was established to deliver culturally-responsive science curricula reflecting both Indigenous knowledge systems and western science traditions [106], and the *Dual-Learning Environment* emerges when scientific and cultural knowledge are held equal to teach Navajo students about stars and planetary system formation, for example [107]. Other concepts have surfaced to acknowledge diverse knowledge systems and work in the space between the two, such as Cree scholar Willie Ermine's *Ethical Space* [108]. The *Negotiated Space*, which focuses on Maori and Western scientific knowledges, describes a conceptual space between different worldviews to explore a topic [109]. The *Both Ways* philosophy, which applies to education, but could also be used in research, focuses on bringing together two forces where tensions or creativity can occur [110,111]. *Both Ways* is said to have evolved from metaphors offered by the Yolgnu people of North East Arnhem land and Warlpiri people of Central Australia. This philosophy brings together "Indigenous Australian traditions of knowledge and Western academic disciplinary positions and cultural contexts, and embraces values of respect, tolerance and diversity" [110].

Conclusion

This scoping review sought to better understand how E/TES is being used in Indigenous health research with the goal of encouraging more thoughtful engagement with and use of the guiding principle. While researchers reported using E/TES in a variety of ways, many did not provide sufficient detail about how they used E/TES in their respective studies. We echo calls made by Elder Dr. Albert Marshall and Dr. Cheryl Bartlett for researchers to intentionally use E/TES in their studies and to clearly articulate this in resulting publications and presentations. We echo these calls not simply to highlight the *usefulness* of E/TES for research, although we believe that to be the case; we argue that E/TES is *needed* to bring together epistemic

communities (i.e., diverse ways of knowing) to foster a new consciousness that more fulsomely explores human participation in the web of life.

Supporting information

S1 Checklist. Preferred Reporting Items for Systematic reviews and Meta-Analyses extension for Scoping Reviews (PRISMA-ScR) checklist.
(DOCX)

S1 Table. Characteristics of articles that report using Etuaptmumk/Two-Eyed Seeing. Year of publication, study participants, geographical location, research design and aim of study.
(DOCX)

S1 Protocol. Protocol for two-eyed seeing scoping review.
(DOCX)

S1 File.
(DOCX)

Acknowledgments

We would like to thank Elder Dr. Albert Marshall and Dr. Cheryl Bartlett for their generosity, patience, kindness, and commitment to sharing and teaching about their work about Two-Eyed Seeing with us. Wela'lioq for continually broadening and enriching our understandings of the guiding principle. Wela'lioq to Elder Dr. Albert for sharing Mi'kmaw knowledge to pass on from generation to generation.

Author contributions

Conceptualization: Anita C. Benoit.

Data curation: Ziwa Yu, Anita C. Benoit.

Formal analysis: Sophie I. G. Roher, Debbie H. Martin, Anita C. Benoit.

Funding acquisition: Debbie H. Martin, Anita C. Benoit.

Investigation: Sophie I. G. Roher, Tara Pride, Marni Amirault, Jenny R. Rand, Anita C. Benoit.

Methodology: Anita C. Benoit.

Project administration: Sophie I. G. Roher, Debbie H. Martin, Anita C. Benoit.

Resources: Ziwa Yu, Anita C. Benoit.

Software: Ziwa Yu, Anita C. Benoit.

Supervision: Debbie H. Martin, Anita C. Benoit.

Validation: Sophie I. G. Roher, Debbie H. Martin, Tara Pride, Marni Amirault, Jenny R. Rand, Anita C. Benoit.

Visualization: Sophie I. G. Roher, Debbie H. Martin, Anita C. Benoit.

Writing – original draft: Sophie I. G. Roher, Debbie H. Martin, Anita C. Benoit.

Writing – review & editing: Sophie I. G. Roher, Debbie H. Martin, Tara Pride, Marni Amirault, Jenny R. Rand, Anita C. Benoit.

References

1. Roher SIG, Yu Z, Martin DH, Benoit AC. How is Etuaptmumk/Two-Eyed Seeing characterized in Indigenous health research? A scoping review. PLoS One. 2021;16: e0254612. https://doi.org/10.1371/journal.pone.0254612 PMID: 34283831

2. Hatcher A, Bartlett C, Marshall M, Marshall A. Two-Eyed Seeing: A cross-cultural science journey. Green Teacher. 2009;86: 3–6.
3. Marshall M, Marshall A, Bartlett C. Two-eyed seeing in medicine. 2nd ed. In: Greenwood M, de Leeuw S, Lindsay NM, editors. Determinants of indigenous Health: Beyond the Social. 2nd ed. Canadian Scholars Press; 2018.
4. Bartlett C, Marshall M, Marshall A, Iwama M. Integrative Science and Two-Eyed Seeing: Enriching the Discussion Framework for Healthy Communities. In: Hallstrom LK, Guehlstorf NP, Parkes MW, editors. Ecosystems Society and Health: Pathways Through Diversity, Convergence, and Integration. Montreal and Kingston: McGill-Queen's University Press; 2015. pp. 280–326. Available: http://www.integrative-science.ca/uploads/articles/2012-Bartlett-Marshall-Iwama-Integrative-Science-Two-Eyed-Seeing-enriching-discussion-framework(authors-draft).pdf
5. Marshall A, Knockwood C, Bartlett C. The Best of Both Worlds. EdCan Network. May 2018: 1–11.
6. Roher SIG, Yu Z, Benoit A, Martin D. Limits and Possibilities: Understanding and Conveying Two-Eyed Seeing Through Conventional Academic Practices. Healthy Populations Journal. 2022;2: 8–13. https://doi.org/10.15273/hpj.v2i2.11295
7. Marshall A. Learning Together by Learning to Listen to Each Other. EdCan Network. May 2018: 1–5. Available: https://www.edcan.ca/articles/learning-together-learning-listen/
8. Marshall A, Bartlett C. Personal communication. 2020.
9. Huria T, Palmer SC, Pitama S, Beckert L, Lacey C, Ewen S, et al. Consolidated criteria for strengthening reporting of health research involving indigenous peoples: The CONSIDER statement. BMC Med Res Methodol. 2019;19: 1–9. https://doi.org/10.1186/s12874-019-0815-8 PMID: 31399058
10. Peters M, Godfrey C, McInerney P, Munn Z, Tricco A, Khalil H. Scoping Reviews. In: Aromataris E, Munn Z, editors. JBI Manual for Evidence Synthesis. 2020. https://doi.org/10.46658/JBIMES-20-12
11. Arksey H, O'Malley L. Scoping studies: Towards a methodological framework. International Journal of Social Research Methodology: Theory and Practice. 2005. https://doi.org/10.1080/1364557032000119616
12. Levac D, Colquhoun H, O'Brien KK. Scoping studies: Advancing the methodology. Implementation Science. 2010. https://doi.org/10.1186/1748-5908-5-69 PMID: 20854677
13. Tricco AC, Lillie E, Zarin W, O'Brien KK, Colquhoun H, Levac D, et al. PRISMA extension for scoping reviews (PRISMA-ScR): Checklist and explanation. Annals of Internal Medicine. 2018. https://doi.org/10.7326/M18-0850 PMID: 30178033
14. Canadian Institutes of Health Rersearch. Defining Indigenous Health Research. 2019 [cited 23 Jan 2019]. Available: http://www.cihr-irsc.gc.ca/e/50340.html
15. Institute for Integrative Sciences and Health. Guiding Principles (Two-Eyed Seeing). 2004 [cited 30 Apr 2018]. Available: http://www.integrativescience.ca/Principles/TwoEyedSeeing/
16. Creswell JW, Plano Clark VL. Designing and Conducting Mixed Methods Research. 3rd ed. Los Angeles: SAGE; 2018.
17. Baydala L, Fletcher F, Tremblay M, Rabbit N, Louis J, Ksay-yin K, et al. A community-university approach to substance abuse prevention. J Community Engagem Scholarsh. 2016;9: 68–76.
18. Benoit AC, Cotnam J, O'Brien-Teengs D, Greene S, Beaver K, Zoccole A, et al. Racism experiences of urban indigenous women in Ontario, Canada: "We all have that story that will break your heart." Int Indig Policy J. 2019. https://doi.org/10.18584/iipj.2019.10.2.1
19. Cabrera LY, Beattie BL, Dwosh E, Illes J. Converging approaches to understanding early onset familial Alzheimer disease: A First Nation study. SAGE Open Med. 2015;3. https://doi.org/10.1177/2050312115621766 PMID: 27092264
20. Latimer M, Sylliboy JR, MacLeod E, Rudderham S, Francis J, Hutt-macleod D, et al. Creating a safe space for First Nations youth to share their pain. Pain Rep. 2018;3: 1–12. https://doi.org/10.1097/PR9.0000000000000682 PMID: 30324174
21. Whitty-Rogers J, Caine V, Cameron B. Aboriginal women's experiences with gestational diabetes mellitus a participatory study with Mi'kmaq women in Canada. Advances in Nursing Science. 2016;39: 181–198. https://doi.org/10.1097/ANS.0000000000000115 PMID: 27149230
22. Vukic A, Gregory D, Martin-misener R, Etowa J. Perspectives for conducting Indigenous qualitative research from a project exploring Mi'kmaw youth mental health. J Ethnogr Qual Res. 2016;10: 209–229.
23. Crooks C V, Lapp A, Auger M, van der Woerd K, Snowshoe A, Rogers BJ, et al. A Feasibility Trial of Mental Health First Aid First Nations: Acceptability, Cultural Adaptation, and Preliminary Outcomes. Am J Community Psychol. 2018;61: 459–471. https://doi.org/10.1002/ajcp.12241 PMID: 29577326

24. Rand JR. Inuit women's stories of strength: Informing Inuit community-based HIV and STI prevention and sexual health promotion programming. Int J Circumpolar Health. 2016;75. https://doi.org/10.3402/ijch.v75.32135 PMID: 27938640
25. Ahmed F, Liberda EN, Solomon A, Davey R, Sutherland B, Tsuji LJS. Indigenous Land-Based Approaches to Well-Being: The Amisk (Beaver) Harvesting Program in Subarctic Ontario, Canada. Int J Environ Res Public Health. 2022;19. https://doi.org/10.3390/ijerph19127335 PMID: 35742603
26. Ahmed F, Liberda EN, Solomon A, Davey R, Sutherland B, Tsuji LJS. Indigenous Land-Based Approaches to Well-Being: The Niska (Goose) Harvesting Program in Subarctic Ontario, Canada. Int J Environ Res Public Health. 2023;20. https://doi.org/10.3390/ijerph20043686 PMID: 36834382
27. Webkamigad S, Warry W, Blind M, Jacklin K. An Approach to Improve Dementia Health Literacy in Indigenous Communities. J Cross Cult Gerontol. 2020;35: 69–83. https://doi.org/10.1007/s10823-019-09388-2 PMID: 31853783
28. Barker M, Uuttuvak A, Lund C, Dragonetti R, Selby P. Adapting Evidence-Based Tobacco Addiction Treatment for Inuit Living in Ontario: A Qualitative Study of Collaboration and Co-creation to Move From Pan-Indigenous to Inuit-Specific Programming. Int J Indig Health. 2021;16. https://doi.org/10.32799/ijih.v16i2.33119
29. Galway LP, Esquega E, Jones-Casey K. "Land is everything, land is us": Exploring the connections between climate change, land, and health in Fort William First Nation. Soc Sci Med. 2022;1. https://doi.org/10.1016/j.socscimed.2022.114700 PMID: 35007947
30. Jacklin K, Pitawanakwat K, Blind M, O'Connell ME, Walker J, Lemieux AM, et al. Developing the Canadian indigenous cognitive assessment for use with indigenous older anishinaabe adults in Ontario, Canada. Innov Aging. 2020;4. 1https://doi.org/0.1093/geroni/igaa038 PMID: 33072890
31. Poirier B, Neufeld HT. "We Need to Live off the Land": An Exploration and Conceptualization of Community-Based Indigenous Food Sovereignty Experiences and Practices. Int J Environ Res Public Health. 2023;20: 4627. https://doi.org/10.3390/ijerph20054627 PMID: 36901638
32. Snooks G, Nagy R, Timms R, Debassige D, Jodouin K, Quenneville B, et al. Blending Feminist, Indigenous, and Participatory Action Research Methodologies: Critical Reflections from the Northeastern Ontario Research Alliance on Human Trafficking. Feminist Formations. 2021;33. https://doi.org/10.1353/ff.2021.0030
33. Sylliboy JR. Coming Out is Part of the Life Cycle: A Qualitative Study using Two-Eyed Seeing to Understand A Two-Spirits Coming Out Process. Glob Public Health. 2021;17. https://doi.org/10.1080/17441692.2021.1993953 PMID: 34710346
34. VanEvery R, Latimer M, Naveau A. Clinical Strategies to Develop Connections, Promote Health and Address Pain From the Perspectives of Indigenous Youth, Elders, and Clinicians. Frontiers in Pain Research. 2022;3. https://doi.org/10.3389/fpain.2022.857624 PMID: 35634453
35. Vorobyova A, Marante A, Cardinal C, Magagula P, Lyndon S, Braley M, et al. Finding the Balance: Embracing the Two-Eyed Seeing Approach to Understand what Cultural Safety in Care Means to Older Adults Living with HIV. Journal of Applied Gerontology. 2022;41. https://doi.org/10.1177/07334648221100803 PMID: 35582747
36. Ward A, Buffalo L, McDonald C, L'Heureux T, Charles L, Pollard C, et al. Supporting First Nations Family Caregivers and Providers: Family Caregivers', Health and Community Providers', and Leaders' Recommendations. Diseases. 2023;11. https://doi.org/10.3390/diseases11020065 PMID: 37218878
37. Ward A, Buffalo L, McDonald C, L'Heureux T, Charles L, Pollard C, et al. Three Perspectives on the Experience of Support for Family Caregivers in First Nations Communities. Diseases. 2023;11. https://doi.org/10.3390/diseases11010047 PMID: 36975596
38. Latimer M, Sylliboy JR, Francis J, Amey S, Rudderham S, Finley GAllen, et al. Co-creating better healthcare experiences for First Nations children and youth: The FIRST approach emerges from Two-Eyed s eeing. Paediatric and Neonatal Pain. 2020;2. https://doi.org/10.1002/pne2.12024 PMID: 35548261
39. Bruner MW, Lovelace R, Hillier S, Baillie CPT, Bruner BG, Hare K, et al. Indigenous Youth Development through Sport and Physical Activity: Sharing Voices, Stories, and Experiences. Int J Indig Health. 2019;14. https://doi.org/10.32799/ijih.v14i2.32958
40. Marsh TN, Cote-Meek S, Toulouse P, Najavits LM, Young NL. The Application of Two-Eyed Seeing Decolonizing Methodology in Qualitative and Quantitative Research for the Treatment of Intergenerational Trauma and Substance Use Disorders. Int J Qual Methods. 2015;14: 1–13. https://doi.org/10.1177/1609406915618046
41. Wright AL, Ballantyne M, Wahoush O. Caring for indigenous families in the neonatal intensive care unit. Nurs Inq. 2020;27: 1–12. https://doi.org/10.1111/nin.12338 PMID: 32030852

42. Peltier C. An Application of Two-Eyed Seeing: Indigenous Research Methods With Participatory Action Research. Int J Qual Methods. 2018;17: 1–12. https://doi.org/10.1177/1609406918812346

43. Martin DE, Thompson S, Ballard M, Linton J. Two-eyed seeing in research and its absence in policy: Little Saskatchewan First Nation elders' experiences of the 2011 flood and forced displacement. Int Indig Policy J. 2017;8. https://doi.org/10.18584/iipj.2017.8.4.6

44. Kurtz DLM, Mahara S, Cash P, Nyberg J, Moller EP. Indigenous methodology in understanding indigenous nurse graduate transition to practice. Int Indig Policy J. 2017;8. https://doi.org/10.18584/iipj.2017.8.4.9

45. Hatala AR, Pearl T, Bird-Naytowhow K, Judge A, Sjoblom E, Liebenberg L. "I Have Strong Hopes for the Future": Time Orientations and Resilience among Canadian Indigenous Youth. Qual Health Res. 2017;27: 1330–1344. https://doi.org/10.1177/1049732317712489 PMID: 28682711

46. Quinn AL. Experiences and well-being among Indigenous former youth in care within Canada. Child Abuse Negl. 2022;123. https://doi.org/10.1016/j.chiabu.2021.105395 PMID: 34781199

47. Marsh TN, Marsh DC, Najavits LM. The Impact of Training Indigenous Facilitators for a Two-Eyed Seeing Research Treatment Intervention for Intergenerational Trauma and Addiction. Int Indig Policy J. 2020;11. https://doi.org/10.18584/IIPJ.2020.11.4.8623

48. Mantyka-Pringle CS, Jardine TD, Bradford L, Bharadwaj L, Kythreotis AP, Fresque-Baxter J, et al. Bridging science and traditional knowledge to assess cumulative impacts of stressors on ecosystem health. Environ Int. 2017;102: 125–137. https://doi.org/10.1016/j.envint.2017.02.008 PMID: 28249740

49. Hall L. Two-eyed seeing in indigenous addiction research and treatment. In: Greaves L, Poole N, Boyle E, editors. Transforming Addiction: Gender, Trauma, Transdisciplinarity. New York: Routledge; 2015. pp. 69–76.

50. Rowan M, Poole N, Shea B, Mykota D, Farag M, Hopkins C, et al. A scoping study of cultural interventions to treat addictions in Indigenous populations: Methods, strategies and insights from a Two-Eyed Seeing approach. Subst Abuse Treat Prev Policy. 2015;10. https://doi.org/10.1186/s13011-015-0021-6 PMID: 26141750

51. Wright AL, Every R Van, Miller V. Indigenous mothers use of web-and app-based information sources to support healthy parenting and infant health in canada: Interpretive description. JMIR Pediatr Parent. 2021;4. https://doi.org/10.2196/16145 PMID: 34018489

52. Tarasuk J, Sullivan M, Bush D, Hui C, Morris M, Starlight T, et al. Findings among Indigenous participants of the Tracks survey of people who inject drugs in Canada, Phase 4, 2017–2019. Canada Communicable Disease Report. 2021;47. https://doi.org/10.14745/ccdr.v47i01a07 PMID: 33679247

53. Njeze C, Bird-Naytowhow K, Pearl T, Hatala AR. Intersectionality of Resilience: A Strengths-Based Case Study Approach With Indigenous Youth in an Urban Canadian Context. Qual Health Res. 2020;30. https://doi.org/10.1177/1049732320940702 PMID: 32684126

54. Hunt C, Michalak A, Lefkimmiatis C, Johnston E, Macumber L, Jocko T, et al. Exploring concussion awareness in hockey with a First Nations community in Canada. Public Health Nurs. 2018;35: 202–210. https://doi.org/10.1111/phn.12407 PMID: 29667222

55. Wright AL, Gabel CA, Bomberry R, Wahoush O. An Application of Two-Eyed Seeing to Community-Engaged Research With Indigenous Mothers. Int J Qual Methods. 2019;18: 1–7. https://doi.org/10.1177/1609406919866565

56. McMillan LJ, Prosper K. Remobilizing netukulimk: indigenous cultural and spiritual connections with resource stewardship and fisheries management in Atlantic Canada. Rev Fish Biol Fish. 2016;26: 629–647. https://doi.org/10.1007/s11160-016-9433-2

57. Castleden HE, Martin DH, Cunsolo A, Harper S, Hart C. Implementing indigenous and western knowledge systems (Part 2): "You have to take a backseat" and abandon the arrogance of expertise. Int Indig Policy J. 2017;8. https://doi.org/10.18584/iipj.2017.8.4.8

58. Marsh TN, Cote-Meek S, Young NL, Najavits LM, Toulouse P. Indigenous healing and seeking safety: A blended implementation project for intergenerational trauma and substance use disorders. Int Indig Policy J. 2016;7. https://doi.org/10.18584/iipj.2016.7.2.3

59. Black K, McBean E. Increased Indigenous Participation in Environmental Decision-Making: A Policy Analysis for the Improvement of Indigenous Health. Int Indig Policy J. 2016;7. https://doi.org/10.18584/iipj.2016.7.4.5

60. Hatala AR, Njeze C, Morton D, Pearl T, Bird-Naytowhow K. Land and nature as sources of health and resilience among Indigenous youth in an urban Canadian context: A photovoice exploration. BMC Public Health. 2020;20. https://doi.org/10.1186/s12889-020-08647-z PMID: 32312240

61. Latimer M, Simandl D, Finley A, Rudderham S, Harman K, Young S, et al. Understanding the Impact of the Pain Experience on Aboriginal Children's Wellbeing: Viewing Through a Two-Eyed Seeing Lens. First Peoples Child Fam Rev. 2014;9: 22–37. https://doi.org/10.1017/CBO9781107415324.004
62. McKivett A, Hudson JN, McDermott D, Paul D. Two-eyed seeing: A useful gaze in Indigenous medical education research. Med Educ. 2020;54: 217–224. https://doi.org/10.1111/medu.14026 PMID: 31958361
63. Fayed ST, King A, King M, Macklin C, Demeria J, Rabbitskin N, et al. In the eyes of Indigenous people in Canada: exposing the underlying colonial etiology of hepatitis C and the imperative for trauma-informed care. Canadian Liver Journal. 2018;1: 115–129. https://doi.org/10.3138/canlivj.2018-0009 PMID: 35991322
64. Whiting C, Cavers S, Bassendowski S, Petrucka P. Using Two-Eyed Seeing to Explore Interagency Collaboration. The Canadian Journal of Nursing Research. 2018;50: 133–144. https://doi.org/10.1177/0844562118766176 PMID: 29648461
65. Sivertsen N, Harrington A, Hamiduzzaman M. 'Two-eyed seeing': the integration of spiritual care in Aboriginal residential aged care in South Australia. J Relig Spiritual Aging. 2020;32: 149–171. https://doi.org/10.1080/15528030.2019.1669515
66. Wright AL, Jack SM, Ballantyne M, Gabel C, Bomberry R, Wahoush O. How Indigenous mothers experience selecting and using early childhood development services to care for their infants. Int J Qual Stud Health Well-being. 2019;14. https://doi.org/10.1080/17482631.2019.1601486 PMID: 30982415
67. Denny SK, Fanning LM. A Mi'kmaw perspective on advancing salmon governance in Nova Scotia, Canada: Setting the stage for collaborative co-existence. Int Indig Policy J. 2016;7. https://doi.org/10.18584/iipj.2016.7.3.4
68. Hovey RB, Delormier T, McComber AM, Lévesque L, Martin DH. Enhancing Indigenous Health Promotion Research Through Two-Eyed Seeing: A Hermeneutic Relational Process. Qual Health Res. 2017;27: 1278–1287. https://doi.org/10.1177/1049732317697948 PMID: 28682710
69. Auger M. Cultural continuity as a determinant of Indigenous peoples' health: A metasynthesis of qualitative research in Canada and the United States. Int Indig Policy J. 2016;7. https://doi.org/10.18584/iipj.2016.7.4.3
70. Victor J, Shouting M, DeGroot C, Vonkeman L, Brave Rock M, Hunt R. I'taamohkanoohsin (everyone comes together): (Re)connecting Indigenous people experiencing homelessness and addiction to their Blackfoot ways of knowing. Int J Indig Health. 2019;14: 42–59. https://doi.org/10.32799/ijih.v14i1.31939
71. Building More Bridges Team. Building More Bridges: Indigenous leadership in a study assessing the impact of distance to care on markers of quality HIV care in Saskatchewan. Canadian Journal of Rural Medicine. 2023;28. https://doi.org/10.4103/cjrm.cjrm_3_22 PMID: 36629167
72. Vukic A, Gregory D, Martin-Misener R, Vukic A, Gregory D, Martin-Misener R. Indigenous Health Research: Theoretical and Methodological Perspectives. Canadian Journal of Nursing Research (CJNR). 2012;44: 146–161.
73. Hall L, Dell CA, Fornssler B, Hopkins C, Mushquash C, Rowan M. Research as Cultural Renewal: Applying Two-Eyed Seeing in a Research Project about Cultural Interventions in First Nations Addictions Treatment. Int Indig Policy J. 2015;6: 1–15. https://doi.org/10.18584/iipj.2015.6.2.4 PMID: 27867445
74. Carter C, Lapum J, Lavallée L, Schindel Martin L, Restoule JP. Urban First Nations Men: Narratives of Positive Identity and Implications for Culturally Safe Care. Journal of Transcultural Nursing. 2017;28: 445–454. https://doi.org/10.1177/1043659616659348 PMID: 27421876
75. Chatwood S, Paulette F, Baker R, Eriksen A, Hansen KL, Eriksen H, et al. Approaching Etuaptmumk—introducing a consensus-based mixed method for health services research. Int J Circumpolar Health. 2015;74: 27438. https://doi.org/10.3402/ijch.v74.27438 PMID: 26004427
76. Saskamoose J, Bellegarde T, Sutherland W, Pete S, McKay-McNabb K. Miýo-pimātisiwin Developing Indigenous Cultural Responsiveness Theory (ICRT): Improving Indigenous Health and Well-Being. Int Indig Policy J. 2017;8. https://doi.org/10.18584/iipj.2017.8.4.1
77. Marsh TN, Coholic D, Cote-Meek S, Najavits LM. Blending Aboriginal and Western healing methods to treat intergenerational trauma with substance use disorder in Aboriginal peoples who live in Northeastern Ontario, Canada. Harm Reduct J. 2015;12: 14. https://doi.org/10.1186/s12954-015-0046-1 PMID: 25989833

78. Marsh TN, Marsh DC, Ozawagosh J, Ozawagosh F. The Sweat Lodge Ceremony: A Healing Intervention for Intergenerational Trauma and Substance Use. The International Indigenous Policy Journal. 2018;9. https://doi.org/10.18584/iipj.2018.9.2.2

79. Hutt-MacLeod D, Rudderham H, Sylliboy A, Sylliboy-Denny M, Liebenberg L, Denny JF, et al. Eskasoni First Nation's transformation of youth mental healthcare: Partnership between a Mi'kmaq community and the ACCESS Open Minds research project in implementing innovative practice and service evaluation. Early Interv Psychiatry. 2019;13: 42–47. https://doi.org/10.1111/eip.12817 PMID: 31243913

80. Blangy S, Bernier M, Bhiry N, Jean-Pierre D, Aenishaenslin C, Bastian S, et al. OHMi-Nunavik: A Multi-Thematic and Cross-Cultural Research Program Studying the Cumulative Effects of Climate and Socio-Economic Changes on Inuit Communities. Ecoscience. 2018;25: 311–324. https://doi.org/10.1080/11956860.2018.1542783

81. Gresku D, Jones C, Donna DL. Collaborative Evaluation Frameworks for Indigenous-Led Community Health Interventions: A Narrative Review. Canadian Journal of Program Evaluation. 2022;37. https://doi.org/10.3138/cjpe.71349

82. Sam J, Richardson CG, Currie LM. Application of Two-Eyed Seeing in Adolescent Mental Health to Bridge Design Thinking and Indigenous Collective Storytelling. Int J Environ Res Public Health. 2022;19. https://doi.org/10.3390/ijerph192214972 PMID: 36429691

83. Stelkia K, Beck L, Manshadi A, Fisk AJ, Adams E, Browne AJ, et al. Letsemot, "Togetherness": Exploring How Connection to Land, Water, and Territory Influences Health and Wellness with First Nations Knowledge Keepers and Youth in the Fraser Salish Region of British Columbia. Int J Indig Health. 2020;16. https://doi.org/10.32799/IJIH.V16I2.33206

84. Wekerle C, McQueen KCD, Barker B, Acai A, Smith S, Allice I, et al. Indigenous Service Provider Perspectives of an Online Education Module to Support Safe Clinical Encounters about Family Violence in Canada. Int J Environ Res Public Health. 2022;19. https://doi.org/10.3390/ijerph192316061 PMID: 36498135

85. Pilarinos A, Field S, Vasarhelyi K, Hall D, Fox ED, Price ER, et al. A qualitative exploration of Indigenous patients' experiences of racism and perspectives on improving cultural safety within health care. CMAJ Open. 2023;11. https://doi.org/10.9778/cmajo.20220135 PMID: 37130609

86. Shrivastava R, Campeau R, Couturier Y, Torrie J, Girard F, Marie-Pierre B, et al. Anthropological perspectives on Miyupimaatisiiun and the integration of oral health in primary care in the Cree communities of Northern Quebec. PLoS One. 2020;15: 1–14. https://doi.org/10.1371/journal.pone.0231406 PMID: 32271846

87. Wright AL, Jack SM, Ballantyne M, Gabel C, Bomberry R, Wahoush O. Indigenous mothers' experiences of using acute care health services for their infants. J Clin Nurs. 2019;28: 3935–3948. https://doi.org/10.1111/jocn.15034 PMID: 31410925

88. Wright AL, Jack SM, Ballantyne M, Gabel C, Bomberry R, Wahoush O. Indigenous mothers' experiences of using primary care in Hamilton, Ontario, for their infants. Int J Qual Stud Health Well-being. 2019. https://doi.org/10.1080/17482631.2019.1600940 PMID: 31033431

89. Danto D, Walsh R, Sommerfeld J. Learning from Those Who Do: Land-Based Healing in a Mushkegowuk Community. Int J Ment Health Addict. 2021;19. https://doi.org/10.1007/s11469-020-00306-z

90. Roburn S. Infrastructure that Sings: Kwawaka'wakw Social Media for Wild Salmon in the Broughton Archipelago. Canadian Journal of Communication. 2021;46. https://doi.org/10.22230/cjc.2021v46n2a3835

91. VanEvery R, Wright A. Mothers' experiences of supporting the healthy development of their infants' indigenous identities. Journal of Indigenous Wellbeing. 2021;6: 82–95.

92. Shrivastava R, Couturier Y, Girard F, Papineau L, Emami E. Two-eyed seeing of the integration of oral health in primary health care in Indigenous populations: a scoping review. Int J Equity Health. 2020;19. https://doi.org/10.1186/s12939-020-01195-3 PMID: 32605562

93. Fontaine LS, Wood S, Forbes L, Schultz ASH. Listening to First Nations women' expressions of heart health: mite achimowin digital storytelling study. Int J Circumpolar Health. 2019;78. https://doi.org/10.1080/22423982.2019.1630233 PMID: 31199204

94. Venner KL, Donovan DM, Campbell ANC, Wendt DC, Rieckmann T, Radin SM, et al. Future directions for medication assisted treatment for opioid use disorder with American Indian/Alaska Natives. Addictive Behaviors. 2018;86: 111–117. https://doi.org/10.1016/j.addbeh.2018.05.017 PMID: 29914717

95. Butler L, Berry L, Exner-Pirot H. Conceptualizing the Role of a Strategist for Outreach and Indigenous Engagement to Lead Recruitment and Retention of Indigenous Students. Nurs Leadersh (Tor Ont). 2018. https://doi.org/10.12927/cjnl.2018.25477 PMID: 29927378

96. Rowett JL. Etuaptmumk: A research approach and a way of being. Antistasis. 2018;1: 54–62.
97. Wolfson L, Poole N, Ninomiya MM, Rutman D, Letendre S, Winterhoff T, et al. Collaborative action on fetal alcohol spectrum disorder prevention: Principles for enacting the truth and reconciliation commission call to action #33. Int J Environ Res Public Health. 2019;16. https://doi.org/10.3390/ijerph16091589 PMID: 31067652
98. Clark WV. Diffusion of personal health practices for Manitoba Inuit. Healthcare Quarterly. 2014;17: 18–23.
99. Kovach M. Indigenous methodologies: Characteristics, Conversations and Contexts. Toronto: University of Toronto Press; 2009.
100. Smith LT. Decolonizing Methodologies: Research and Indigenous Peoples. New York. London: Zed Books; 1999.
101. Wilson S. Research is Ceremony. Black Point: Fernwood Pub; 2008.
102. Simonds VW, Christopher S. Adapting western research methods to indigenous ways of knowing. Am J Public Health. 2013;103: 2185–2192. https://doi.org/10.2105/AJPH.2012.301157 PMID: 23678897
103. Knowledge Gardening. In: Institute for Integrative Science and Health [Internet]. 2005 [cited 22 Jul 2024]. Available: http://www.integrativescience.ca/Themes/Knowledge/
104. Gray MA, Walker EM, Brooks C, DeMarsh L. Analysis of Environmental Contaminants in Muskrat Root, Acorus americanus Raf.: A Traditional Indigenous Medicinal Plant. Bull Environ Contam Toxicol. 2019;102: 275–279. https://doi.org/10.1007/s00128-018-2510-6 PMID: 30552440
105. "Strong Like Two People": A photographer's ode to First Nations' special relationship with the land in Canada. In: True North Photo Journal [Internet]. [cited 22 Jul 2024]. Available: https://truenorthjournal.ca/2017/06/on-the-land/
106. Stephens S. Handbook for Culturally Responsive Science Curriculum. Fairbanks; 2000. Available: http://www.ankn.uaf.edu/curriculum/Articles/BarnhardtKawagley/Indigenous_Knowledge.html
107. Astrobiology at NASA. NASA and the Navajo Nation. NASA. 2018. Available: https://astrobiology.nasa.gov/education/nasa-and-the-navajo-nation/
108. Ermine W. The Ethical Space of Engagement. Indigenous Law Journal. 2007;6: 193–203. https://doi.org/10.3868/s050-004-015-0003-8
109. Mila-Schaaf K, Hudson M. The interface between cultural understandings: negotiating new spaces for Pacific mental health The interface between cultural understandings: Negotiating new spaces for Pacific mental health Author information. Pac Health Dialog. 2009;15: 113–119.
110. Hall L, Maughan C, Wilkes M, Forrest J, Harrison A. Swimming not drowning–resilience as a key determinant of success for Aboriginal and Torres Strait Islander pre-tertiary students. Journal for Multicultural Education. 2015;9: 159–173. https://doi.org/10.1108/JME-12-2014-0045
111. Ober R. Reflections on both-ways at Batchelor institute. BiiteN. 2004;13: 8–9. Available: https://search.informit.org/doi/abs/10.3316/informit.969785550033760
